# Seizures associated with antibodies against cell surface antigens are acute symptomatic and not indicative of epilepsy: insights from long-term data

**DOI:** 10.1007/s00415-020-10250-6

**Published:** 2020-10-06

**Authors:** Anna Rada, Robert Birnbacher, Claudio Gobbi, Martin Kurthen, Albert Ludolph, Markus Naumann, Ulrike Neirich, Tim J. von Oertzen, Gerhard Ransmayr, Matthias Riepe, Mareike Schimmel, Oliver Schwartz, Rainer Surges, Christian G. Bien

**Affiliations:** 1 Epilepsy Center Bethel, Krankenhaus Mara, Epilepsy Centre Bethel, Krankenhaus Mara, Maraweg 17-21, 33617 Bielefeld, Germany; 2 Department of Pediatrics and Adolescent Medicine, Villach General Hospital, Villach, Austria; 3 Department of Neurology, Neurocenter of Southern Switzerland (NSI), 6900 Lugano, Switzerland; 4 Faculty of Biomedical Sciences, Università Della Svizzera Italiana (USI), 6900 Lugano, Switzerland; 5 Swiss Epilepsy Center, Zürich, Switzerland; 6 Department of Neurology, University of Ulm, Ulm, Germany; 7 Department of Neurology and Clinical Neurophysiology, University of Augsburg, Augsburg, Germany; 8 Department of Pediatrics, Neurology, Stiftungskrankenhäuser Frankfurt Am Main, Clementine Kinderhospital, Frankfurt am Main, Germany; 9 Department of Neurology 1, Kepler University Hospital GmbH, Johannes Kepler University Linz, Linz, Austria; 10 Department of Neurology 2, Kepler University Hospital GmbH, Johannes Kepler University Linz, Linz, Austria; 11 Division of Gerontopsychiatry, Ulm University, Günzburg, Germany; 12 Department of Pediatrics, Section of Neuropediatrics, University of Augsburg, Augsburg, Germany; 13 Department of Pediatric Neurology, Münster University Hospital, Münster, Germany; 14 Department of Epileptology, University Hospital of Bonn, Bonn, Germany; 15 Laboratory Krone, Bad Salzuflen, Germany

**Keywords:** Autoimmune encephalitis, Neural antibodies, Acute symptomatic seizures, Epilepsy, Long-term course

## Abstract

**Background:**

Clinicians have questioned whether any disorder involving seizures and neural antibodies should be called “(auto)immune epilepsy.” The concept of “acute symptomatic seizures” may be more applicable in cases with antibodies against neural cell surface antigens. We aimed at determining the probability of achieving seizure-freedom, the use of anti-seizure medication (ASM), and immunotherapy in patients with either constellation. As a potential pathophysiological correlate, we analyzed antibody titer courses.

**Methods:**

Retrospective cohort study of 39 patients with seizures and neural antibodies, follow-up ≥ 3 years.

**Results:**

Patients had surface antibodies against the *N*-methyl-d-aspartate receptor (NMDAR, *n* = 6), leucine-rich glioma inactivated protein 1 (LGI1, *n* = 11), contactin-associated protein-2 (CASPR2, *n* = 8), or antibodies against the intracellular antigens glutamic acid decarboxylase 65 kDa (GAD65, *n* = 13) or Ma2 (*n* = 1). Patients with surface antibodies reached first seizure-freedom (88% vs. 7%, *P* < 0.001) and terminal seizure-freedom (80% vs. 7%, *P* < 0.001) more frequently. The time to first and terminal seizure-freedom and the time to freedom from ASM were shorter in the surface antibody group (Kaplan–Meier curves: *P* < 0.0001 for first seizure-freedom; *P* < 0.0001 for terminal seizure-freedom; *P* = 0.0042 for terminal ASM-freedom). Maximum ASM defined daily doses were higher in the groups with intracellular antibodies. Seizure-freedom was achieved after additional immunotherapy, not always accompanied by increased ASM doses. Titers of surface antibodies but not intracellular antibodies decreased over time.

**Conclusion:**

Seizures with surface antibodies should mostly be considered acute symptomatic and transient and not indicative of epilepsy. This has consequences for ASM prescription and social restrictions. Antibody titers correlate with clinical courses.

**Electronic supplementary material:**

The online version of this article (10.1007/s00415-020-10250-6) contains supplementary material, which is available to authorized users.

## Introduction

In its most recent classification paper, the International League Against Epilepsy (ILAE) introduced the new etiological category of “immune epilepsy.” The ILAE herewith referred to the emerging group of autoimmune encephalitides and explicitly mentioned antibodies against the *N*-methyl-d-aspartate receptor (NMDAR) and leucine-rich glioma inactivated protein 1 (LGI1) [40]. Autoimmune conditions with seizures have frequently been studied under the heading “autoimmune epilepsy” [3, 31, 39]. Recently, researchers [24] and the Autoimmunity/Inflammation Task Force [44] of the ILAE have suggested that patients with autoimmune encephalitides and pathogenic antibodies against cell surface antigens should be considered to have “seizures secondary to autoimmune encephalitis” in the sense of acute symptomatic seizures [1], should not regularly receive long-term anti-seizure medication (ASM), and be exempt from the social restrictions for epilepsy patients. In contrast, autoimmune-related seizures that recur in an unprovoked manner and are resistant to immunological therapy should be called “autoimmune-associated epilepsy.” These conditions are T cell-driven encephalitides [6], often with antibodies against intracellular antigens like glutamic acid decarboxylase 65 kDa (GAD65) [9], onconeural proteins [42] or Rasmussen encephalitis [50]. There are unfortunate cases with surface antibodies who also develop “autoimmune-associated epilepsy.” Geis and colleagues tentatively suggested one year as a cut-off between seizures secondary to autoimmune encephalitis and autoimmune epilepsy [24]; the ILAE task force felt that the database for such a border was still insufficient [44]. Here, we present long-term outcome data that support such a general distinction, but with a much longer time frame to become seizure-free for the group of patients with autoimmune encephalitides and surface antibodies.

## Methods

### Patients

We included patients of any age if they: (1) harbored neural antibodies in serum or CSF (CSF antibodies were needed for the diagnosis of NMDAR antibodies [25], serum titers ≥ 1:128 were required for contactin-associated protein-2 (CASPR2) antibodies [4], and ≥ 1:1500 for GAD65 antibodies [15]); (2) had > 1 seizure; (3) a follow-up of ≥ 36 months; and (4) at least three antibody titers (serum or CSF).

CGB identified the patients from the databases of the Bethel Antibody Laboratory and the Laboratory Krone (2011–2019). The treating physicians offered repeated consultations and examinations, including antibody tests for clinical reasons and the patients utilized these services. The responsible physicians retrospectively collected clinical information. AR and CGB extracted the following data: seizure frequency or occurrence of seizures (yes/no); ASM defined daily doses (DDD); time to antibody diagnosis and time to start of immunotherapy; types and numbers of immunotherapies and their duration. AR and CGB rated clinical performance by consensus according to the modified Rankin Scale (mRS) [26]. Values ≤ 2 indicate independent living of the patient and values > 2 increasing degrees of dependency. “Seizure-freedom” and “ASM-freedom” required this status for ≥ 12 months. We only counted clinical seizures.

### Methods

Antibodies and their titers (multiples of 1:2) were determined in the Bethel Antibody Laboratory and (since 2016) in the Laboratory Krone, as described previously [2, 4]. Ma2 titers were determined with the tissue-based assay [2, 4].

### Graphs and statistics

**Fig. 1:**
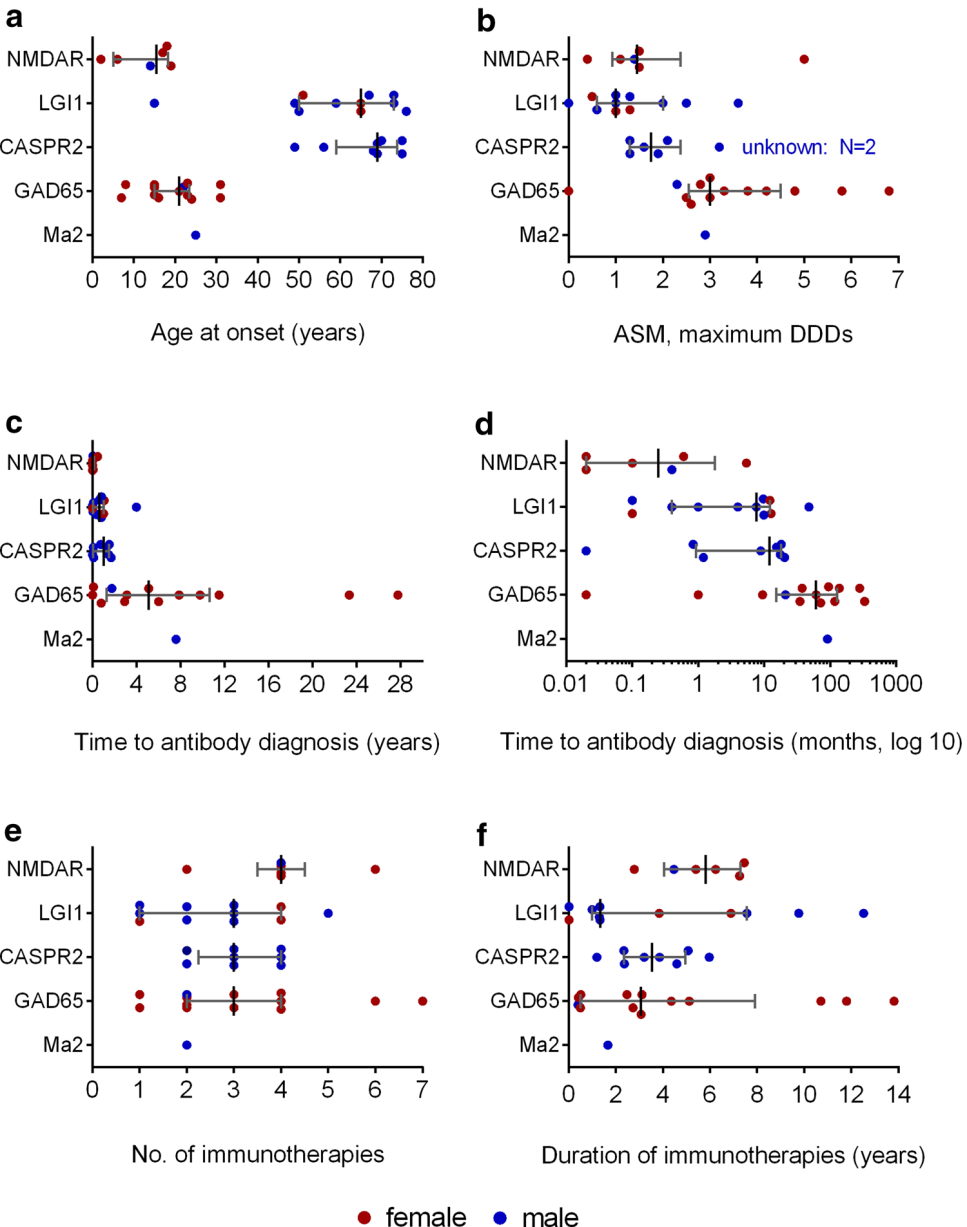
Characteristics of the antibody-defined groups. ASM, anti-seizure medication; CASPR2, contactin-associated protein-2; DDD, defined daily dose; GAD65, glutamic acid decarboxylase 65 kDa; LGI1, leucine-rich glioma inactivated protein 1; NMDAR, N-methyl-d-aspartate receptor. Lines and whiskers indicate medians and interquartile ranges

**Fig. 2:**
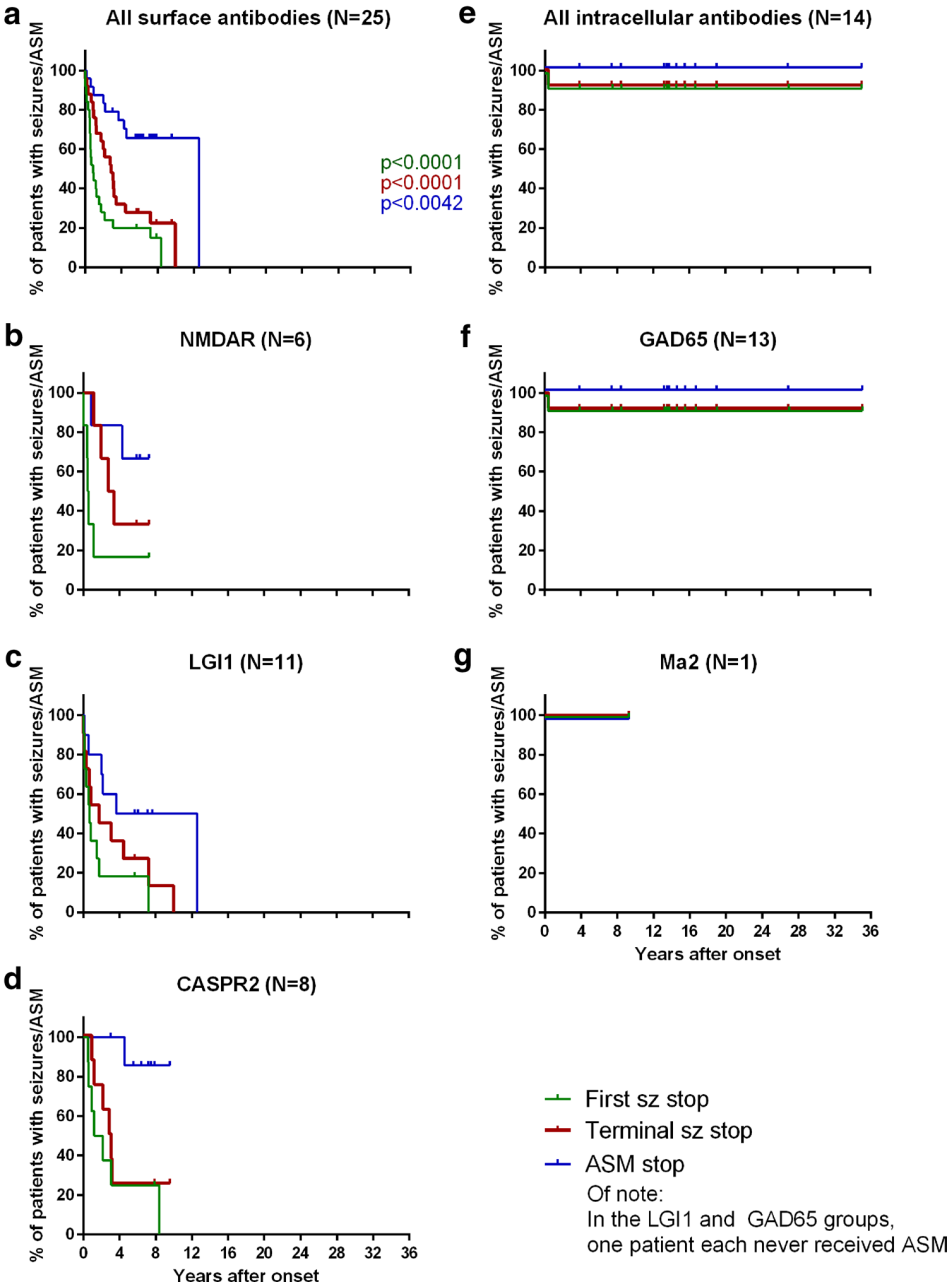
Seizure and anti-seizure medication (ASM) freedom over time. Kaplan-Meier-curves. Lines: censored cases. CASPR2, contactin-associated protein-2; GAD65, glutamic acid decarboxylase 65 kDa; LGI1, leucine-rich glioma inactivated protein 1; NMDAR, *N*-methyl-d-aspartate receptor

Demographic data are presented in Fig. 1. For group-wise comparisons, we used the Mann–Whitney *U* test or the two-tailed Fisher’s exact test (Prism, version 6, GraphPad Software Inc., San Diego, CA). We depicted the times to seizure and ASM-freedom using Kaplan–Meier curves (Fig. 2) and analyzed the differences with Mantel–Cox log-rank tests (Prism). For each patient, we prepared diagrams for the following parameters: seizures, ASM, mRS, immunotherapies, and antibody titers in serum and CSF; they are shown in the Supplementary Figure.

**Fig. 3:**
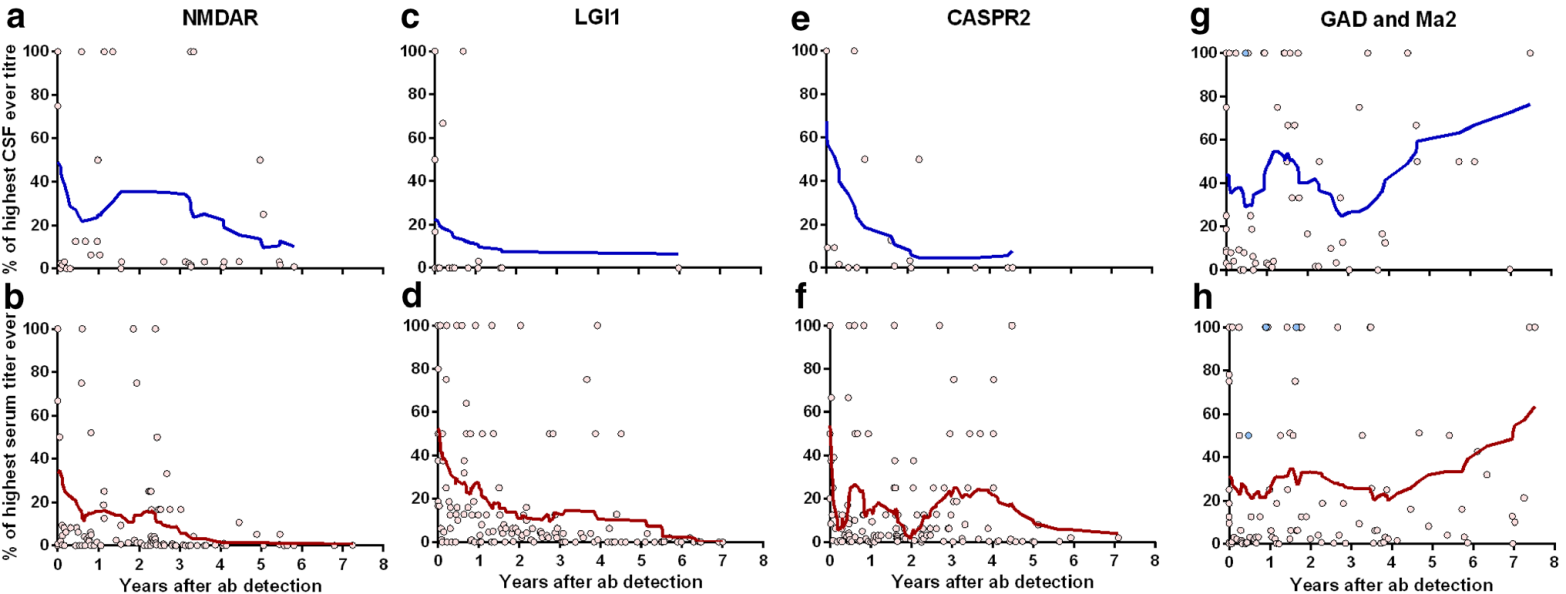
Courses of antibody titers in the cerebrospinal fluid (CSF, upper row, blue) and serum (lower row, red) during the first eight years after disease onset. The dots show all individual values, expressed as a percent of the individual’s highest titer. The blue dots in G and H are those of the patient with Ma2 antibodies. The lines are smoothed averages (see the Methods section)

We preliminarily set the significance level to *P* < 0.05 and used Bonferroni correction for 11 tests. Thus, the significance level was *P* < 0.0045. We averaged antibody titers (expressed as the percent of the individual’s highest recorded titer) using 15 neighboring points with a second order polynomial smoothing (Prism), see Fig. 3.

### Ethics

This study was approved by the Ethics Committee of the University of Münster, Germany (2018–436-f-S). Since this was a retrospective analysis of data from the authors’ own clinical practice, patients’ consent was not required.

## Results

We included 39 patients. The median disease duration from disease manifestation to most recent follow-up was 7.5 years (range 3.0–35.1), and the median time from antibody detection to most recent follow-up was 6.4 years (3.0–13.9 years). The patients harbored antibodies directed against the following antigens: NMDAR, *n* = 6; LGI1, *n* = 11; CASPR2, *n* = 8 (cell surface antigens); GAD65, *n* = 13; Ma2, *n* = 1 (intracellular antigens). The patients’ characteristics are shown in Fig. 1 and Table 1; individual data and information, which patients had been included in previous studies [2, 4–6, 15, 36, 41] are given in Supplementary Table 1.

**Table 1:** Patients’ data

Antigens	No. of males/females (% females)	Age at disease onset [y]	From disease onset to antibody diagnosis [mo after onset]	ASM: max DDD	Time to start of first immuno-tx [mo after onset]	No. of immuno-txs	Duration of immuno-tx [mo]	No. of relapses	From disease onset to last follow-up [mo]	From ab detection to last follow-up [mo]	Duration to first seizure-freedom [mo]	Duration of sz-freedom at most recent follow-up [mo]	No. of terminally sz-free patients	From disease onset to ASM discontinuation [mo]	No. of patients w/o ASM for ≥ 1 y at most recent follow-up	Develop-ment of hippo-campal sclerosis
NMDAR *n* = 6	1/5 (83%)	16 (2–19)	0.3 (0–5.4)	1.5 (0.4–5.0)	0.5 (0–5.9)	4 (2–6)	70 (34–89)	2 (1–5)	78 (68–89)	78 (65–89)	7 (0.1–14)	43 (37–68)	4 (67%)	10.8 and 51.5	2/6	0/6 (0%)
LGI1 *n* = 11	8/3 (27%)	65 (14–76)	7.6 (0.1–47.9)	1.2 (0.5–3.6)	6.1 (0.4–13.2)	3 (1–5)	16 (0–150)	0 (0–2)	84 (59–172)	77 (47–164)	8 (0.2–87)	58 (38–84)	10 (91%)	25.1 (1.7–151)	6/11	7 (2 bilat.)/11 (64%)
CASPR2 *n* = 8	8/0 (0%)	69 (49–75)	12.2 (0–20.3)	1.8 (1.3–3.6)	7.3 (0.6–15.8)	3 (2–4)	42 (14–72)	0 (0–2)	82 (48–115)	63 (47–100)	13 (6–37)	35 (21–79)	6 (75%)	54.9	1/8	1/5^1^ (20%)
GAD65 *n* = 13	1/12 (92%)	21 (7–31)	61.2 (0–331)	3.2 (0–6.8)	35.0 (1.5–336)	3 (1–7)	37 (5–166)	0 (0–0)	165 (36–421)	88 (36–166)	4	32	1 (8%)	–^2^	1/13^2^	4 (2 bilat.)/13 (31%)
Ma2 *n* = 1	1/0 (0%)	24	91.1	2.9	91.2	2	20	0	111	20	Never sz-free	Never sz-free	0	–	0/1	1 (100%)

Medians (ranges)

^1^No information: *n* = 1; no MRI done: *n* = 2

^2^The one patient who became seizure-free was never treated with ASM

*ASM,* anti-seizure medication, *bilat*. bilateral, *CASPR2* contactin-associated protein-2, *DDD* defined daily dose, *GAD65* glutamic acid decarboxylase, *LGI1* leucine-rich glioma inactivated protein 1, *mo* months, *MRI* magnetic resonance imaging, *NMDAR* *N*-methyl-d-aspartate receptor, *No* number, *sz* seizure, *tx* therapy, *w/o* without, *y* year

Demographics were similar to previous publications on surface [10], GAD65 [38], and Ma2 [11] antibodies. All patients with surface antibodies (apart from patient CASPR2-2) had definite autoimmune encephalitides according to recent diagnostic criteria [25], either in the form of definite limbic encephalitis [25] or faciobrachial dystonic seizures [46]. In contrast, only two patients with intracellular antibodies (GAD65-4, -9) started as definite autoimmune encephalitis, both in the form of a limbic encephalitis (*P* < 0.0001). The maximum ASM-DDD were higher in the patients with intracellular antibodies (median 3.0, range 0–5.0) compared to those with surface antibodies (median 1.3, range 0–6.8, *P* = 0.0003). The time from manifestation to antibody diagnosis was longer with intracellular antibodies (median 67, range 0–333 months) compared to surface antibodies (median 4, range 0–48 months, *P* = 0.0001). The following immunotherapies were administered (in brackets: number of treated patients): intravenous methylprednisolone (IVMP) or oral prednisolone (*n* = 37); intravenous immunoglobulins (IVIG, *n* = 15); immunoadsorption (*n* = 22); plasma exchange (*n* = 1); azathioprine (*n* = 17); mycophenolate mofetil (*n* = 11); rituximab (*n* = 7); cyclophosphamide (*n* = 6); natalizumab (*n* = 2); basiliximab (*n* = 1). The groups did not differ in the number of immunotherapies (surface antibodies: median 3, range 1–6; intracellular antibodies: median 2.5, range 1–7; *P* = 0.62) or duration of immunotherapies (surface antibodies: median 3.8, range 0–12.5 years; intracellular antibodies: median 2.9, range 0.4–13.8 years; *P* = 0.57).

### Hippocampal sclerosis

Hippocampal sclerosis (HS), on magnetic resonance imaging (MRI), developed in patients with antibodies against LGI1 (LGI1-2, -4, -5, -6, -7, -9, -11; 64%), GAD65 (GAD65-4, -7, -9, -13; 31%), and CASPR2 (CASPR2-4; 20%), but not with NMDAR antibodies. The patient with Ma2 antibodies underwent an anteromedial temporal lobe resection ten years after disease onset; histologically, he had HS, ILAE type 3 with signs of chronic inflammation. HS had not been diagnosed preoperatively by MRI. A postoperative follow-up is not yet available. The following patients also underwent temporal lobe surgery, with histopathology congruent to MRI diagnoses: GAD65-4, HS (type 3); GAD65-5, no HS; GAD65-9, HS (type 3); GAD-13 HS (type 1). None of them became seizure-free.

### Seizure-freedom, ASM-freedom

At most recent follow-up, the group with surface antibodies was superior regarding first and terminal seizure-freedom (Table 2). Median duration of seizure-freedom at most recent follow-up was 4.4 years (range 1.8–7.0 years). The time to first and terminal seizure-freedom and to ASM-freedom is depicted in Fig. 2 in the form of Kaplan–Meier curves. The patients with surface antibodies did better in all three parameters. Supplementary Table 2 shows the year-wise proportions of seizure-free patients.

**Table 2:** Outcome at most recent follow-up: First and terminal seizure-freedom and anti-seizure medication (ASM) freedom

	First seizure-freedom		Terminal seizure-freedom		ASM-freedom		Total
	Seizure-free	Not seizure-free	Seizure-free	Not seizure-free	ASM stopped or never given	ASM ongoing	
Surface antibodies	22 (88%)	3 (12%)	20 (80%)	5 (20%)	11 (44%)	14 (56%)	25
Intracellular antibodies	1 (7%)	13 (93%)	1 (7%)	13 (93%)	1 (7%)	13 (93%)	14
Sums	23	16	21	18	12	27	39
*P*	< 0.0001		< 0.0001		0.0283 (n.s.)		

*n.s*. not significant

While the patients with intracellular antibodies only exceptionally became seizure-free, seizure frequency decreased over the years by ≤ 50% compared to onset in 8/13 cases (in one case—GAD-7-no information about treatment during improvement was available). The seizure reduction was related to immunotherapy (GAD-12), ASM plus immunotherapy (GAD-4, -9 [plus epilepsy surgery], 10), ASM only (GAD-3, GAD-6) or no new intervention (GAD-1, -2).

Of note, 13/39 patients achieved terminal seizure-freedom after > 1 year (Supplementary Table 1); the maximum lapse was ten years (patient LGI1-9). This was partly due to relapses in the surface group (there were no relapses with intracellular antibodies). During relapses with seizures, semiology was the same as before where sufficient data were available. In 7/25 patients in the surface group, even the time to first seizure-freedom lasted more than one year; five of them continuously had seizures before they achieved first seizure-freedom (median 21 months, range 15–87). When patients became seizure-free, they had received additional immunotherapy but not always more intense ASM (Supplementary Table 3).

### Antibody titer courses

Titer courses started at different heights. The normalized and averaged serum and CSF antibody titers decreased over time in the patients with surface antibodies (exceptions on the individual level were only serum antibodies in patients CASPR2-5, -7,-8), but not in those with intracellular antibodies (Fig. 3).

In many individuals, the titer courses corresponded with fluctuations in seizure frequency and mRS (NMDAR-1, -4 in CSF; NMDAR-1, -4, -5, -6, LGI1-4, -6, -7, CASPR2-3, -7 in serum, partly in the absence of sufficient CSF studies). An elevation in serum LGI1 antibodies heralded the first relapse in patient LGI1-4. In other instances, titers increased again without clinical deterioration (LGI1-2, CASPR2-1, -4, -6, -8, all in serum). Occasionally, serum titers kept decreasing after seizures and elevated mRS had already remitted (LGI1-3, LGI1-11, CASPR2-2, all in serum). Elevated CSF antibodies became unmeasurable in only 2/15 cases with surface antibodies (both LGI1) and 3/13 with intracellular antibodies (all GAD65). Serum titers became undetectable in 7/11 cases with antibodies against LGI1, 2/5 against the NMDAR, 0/8 against CASPR2, 0/13 against GAD65, and 0/1 against Ma2. Apheresis had the strongest immediate effect on titers (NMDAR-2, CASPR2-3, -4 in CSF; LGI1-1, -8, -10, in serum). However, titers often rose again after such interventions, especially if there was insufficient steroid or immunosuppressive treatment after the apheresis (NMDAR-1, -3, -4, -5, LGI1-2, -4, -5, -11, CASPR2-1, -3, -4, -7, -8, all in serum, an exception being LGI1-10).

In most patients with intracellular antibodies, serum and CSF titers did not decrease in the long term. Periods of intense immunotherapy could lower the titers, but they eventually increased again (GAD65-1, -5, 6, -8, 9, 10 in serum; GAD65-2, -3, -10 in serum and CSF). The case of GAD65-12 was exceptional and spectacular. This patient, with recent manifestation of diabetes mellitus type I and Hashimoto thyroiditis, had her first seizure at age 15 years. There were no other features of limbic encephalitis. GAD65 antibodies were detected two weeks later, and she received her first monthly IVMP pulse with another 4 weeks later. Six pulses, five times of 1 g each, were administered. She never received ASM. Seizure frequency reduced. She had her last seizure 2.5 months after the first IVMP pulse. Her titers were strongly reduced.

## Discussion

This retrospective study examined patients with neural autoantibodies and seizures and—as the most interesting novelty—analyzed long-term courses ≥ 3 years (median 7.5 years). Patients with surface antibodies achieved first and terminal seizure-freedom in 88% and 80% of cases. This is the same proportion as the 81% of patients with a seizure in close temporal association with a documented brain insult and without subsequent unprovoked seizures over ten years in a classical study [28]. These seizures were defined by the ILAE as “acute symptomatic” [1]. In contrast, only one patient with intracellular antibodies (7%) reached this favorable outcome (Table 2). The remaining rate of 93% of patients with subsequent unprovoked seizures lies in the range of the > 60% relapse risk that defines epilepsy according to the ILAE [22]. Patients with surface antibodies had lower maximum ASM-DDD (Fig. 1b), and ASM were discontinued earlier (Fig. 2a vs. 2E). Patients could become seizure-free after more than one year, always with additional immunotherapies, but not necessarily with ASM-DDD increases; in fact, the patients LGI1-2, -4, -11 became seizure-free while they were not taking any ASM (Supplementary Table 3). These observations correlate with the decreasing titers of surface but not of intracellular antibodies (Fig. 3). Thus, autoimmune encephalitides with NMDAR, LGI1, or CASPR2 antibodies are ictogenic but not usually epileptogenic. They force the brain to seize as long as the antibodies are present in a sufficiently high concentration but they rarely transform the central nervous system in a way that it generates recurrent unprovoked seizures, i.e., the observed seizures are but symptoms of a transient external disease process [1]. Intracellular antibodies, on the other hand, seem to be markers of a profound, enduring, and ASM-resistant propensity to generate recurrent unprovoked epileptic seizures, i.e., epilepsy [23]. These patients even respond poorly to epilepsy surgery, here and in the literature [8]. Out data thereby confirm previous hypotheses [24, 44]. They refine Geis’ and colleagues' preliminary suggestion that seizures for more than one year in patients with autoimmune encephalitides should lead to the diagnosis of epilepsy. According to our data, it can take up to seven years before continuously recurrent seizures secondary to an autoimmune encephalitis subside.

The term *acute* symptomatic seizures may appear stretched in such long-term courses. These are, however, exceptional in this sample. Whereas the demographic data of our cases were as expected from the antibody types, the long clinical follow-up was special. Delays to seizure-freedom were much longer in our patients than in a previous unselected series that reported on a median lag to seizure remission within a month after start of immunotherapy (interquartile range 0.3–2.4 months) [13]. Our long-term follow-up sample demonstrates the concept at its extremes. At the same time, it shows that terminal seizure-freedom in such cases was long-lasting and stable (≥ 21 months of terminal seizure-free follow-up).

An epilepsy diagnosis results in marked driving restrictions and limited professional abilities. We suggest that patients with seizures secondary to autoimmune encephalitides and antibodies against NMDAR, LGI1, or CASPR2 should be individually evaluated for social restrictions, as suggested by a UK guideline that mentions “limbic encephalitis associated with seizures” together with acute encephalitides and meningitis, apart from chronic epilepsy [16]. Congruently, a review article reported a low long-term risk (< 15%) to develop epilepsy after encephalitides associated with surface antibodies [43].

The time to diagnosis of surface antibodies was shorter compared to intracellular antibodies (NMDAR < LGI1 < CASPR <  < GAD65, Table 1 and Fig. 1d). This phenomenon is probably due to the subacute start in the autoimmune encephalitides with surface antibodies [13], which is only occasionally noted in patients with GAD65 antibodies and a limbic encephalitis at onset [37].

Individual titer courses of surface antibodies, to some extent, moved in parallel to the clinical courses. In some cases, antibodies increased again despite clinical recovery, or antibodies fell more slowly than patients recovered—especially CASPR2 antibodies. Antibody titers, thus, did not in general predict the immediately subsequent clinical course. Titers of intracellular antibodies remained high or increased again after transient reduction by intense immunotherapies. Titer courses confirmed chronicity but were otherwise not clinically informative.

HS occurred at similar frequencies in patients with surface and intracellular antibodies. It occurred most frequently with LGI1 antibodies (64%), despite the favorable seizure outcome in this group. This proportion is in line with existing figures of 41% [49] or 50% [21]. Other studies reported hippocampal abnormalities with NMDAR antibodies in < 10%, [36, 37] again congruent with our data (0%) [20, 30]. CASPR2 antibodies (20%) and GAD65 antibodies (31%) were between those values, again consistent with the literature reporting on 20–24% [32, 48] and 33–62% [17, 35] of cases, respectively, having hippocampal lesions or atrophy/sclerosis. Hence, in patients with neural antibodies, obvious hippocampal damage is neither necessary nor sufficient for the development of epilepsy. A caveat comes from the case with Ma2 antibodies, whose HS was only diagnosed under microscope. The true frequency of structural epileptogenic damage might be underestimated in this series.

Immunotherapy is the most relevant treatment in seizures secondary to autoimmune encephalitis. Recurrent seizures and cognitive decline may be prevented if immunotherapy is applied early in patients with surface antibodies [7, 13, 29, 46, 47] but not with intracellular antibodies [7, 36]. This hypothesis is confirmed by the present series. The first-line and second-line therapy concept derived from the retrospective analysis of treatment courses in anti-NMDAR encephalitis [12] has been widely adopted in autoimmune encephalitides with different surface antibodies [7, 33]. It can be recognized in cases of the present series, including those with anti-NMDAR encephalitis who only responded to second-line therapy with rituximab. Most patients had several immunological treatment approaches. Due to the retrospective and uncontrolled documentation of combination therapies, we did not attempt to disentangle the contribution of single interventions.

Patients with intracellular antigens did not benefit from immunotherapy, except for one patient (GAD65-12) who became seizure-free under IVMP pulses starting six weeks after disease onset. A similar case has recently been described [14]. In contrast, immunotherapy does not stop seizures in chronic patients with GAD65 antibodies [36].

Plasmapheresis or immunoadsorption had an immediate but no long-lasting effect on antibody courses. A special case was patient LGI1-10, who stopped having seizures and had reduced antibody titers after only one sequence of immunoadsorption.

ASMs are mostly considered as an add-on-therapy for patients with autoimmune encephalitides with surface antibodies [18], but usually not as a stand-alone or long-term treatment [13, 34]. They usually do not have a strong effect unless applied together with immunotherapy [19, 46]. This can also be observed in our sample: Only 1/11 cases, in which ASM was discontinued, had a seizure relapse (NMDAR-1, latency 2 days, 14 months after disease onset; immunotherapy had been stopped 3.5 months before, and there were still NMDAR antibodies in serum and CSF). Vice versa, only 1/3 cases with introduction of an ASM without a parallel intensification or introduction of immunotherapy experienced a reduction in seizure frequency (NMDAR-5, four years after onset); in the other two instances, mere introduction of ASM was without effect on seizure frequency (LGI1-7, 4.5 years after onset; CASPR2-4, 3.5 years after onset, both with HS).

This study has limitations. First, we documented and rated data retrospectively, a process that has inherent problems. Second, the study group is biased toward difficult-to-treat patients. Third, patients were treated by various ASM and immunotherapies in different orders and combinations. Fourth, the patients did not systematically undergo prolonged video-EEG monitoring to capture unnoticed or unaware seizures [45]. Fifth, the data do not directly determine *ante hoc* in an individual patient with surface antibodies when to diagnose an “epilepsy.” One may tentatively suggest to diagnose epilepsy if seizures go on for more than one year even though the antibodies in serum (LGI1 or CASPR2) or CSF (NMDAR) [27] have gone down by more than three or more than titer levels compared to onset, especially (but not necessarily), if potentially epileptogenic atrophic brain damage is evident. This phenomenon seems to be the case for these four patients: LGI1-7, CASPR2-4 (both with HS), and NMDAR-3, -5 (without structural damage). After symptom control, immunotherapies could be discontinued without early deteriorations, regardless of titers (NMDAR-6, LGI1-1, -3, -5, -7 [epilepsy persisted], -8, -9, 10, CASPR2-1, -4 [epilepsy persisted],-5, -6; two of these patients relapsed after > 1 year: NMDAR-6; LGI1-9). ASM contributed less than immunotherapies; they could be discontinued without provoking a seizure relapse (the only exception being NMDAR-1).

In conclusion, this study shows, on a group level, that patients with autoimmune encephalitides and surface antibodies have acute symptomatic seizures that do usually not require long-term immunological or ASM therapy or social restrictions. In contrast, patients with intracellular antibodies and seizures—if not treated early on—develop epilepsy. Antibody titers can partially help to interpret the clinical courses, especially on the group level.

## Electronic supplementary material

Below is the link to the electronic supplementary material.

Supplementary file1 (PDF 760 kb)

## Data Availability

Data will be shared upon request from any qualified investigator, while maintaining anonymization of the patients.

## Abbreviations

ASM: Anti-seizure medication
CASPR2: Contactin-associated protein-2
DDD: Defined daily doses
GAD65: Glutamic acid decarboxylase 65 kDa
HS: Hippocampal sclerosis
ILAE: International league against epilepsy
LGI1: Leucine-rich glioma inactivated protein 1
NMDAR: *N*-Methyl-d-aspartate receptor
IVMP: Intravenous methylprednisolone
