# Supplementary material for: Seizures associated with antibodies against cell surface antigens are acute symptomatic and not indicative of epilepsy: insights from long-term data
Source: J Neurol. 2020 Oct 6;268(3):1059–69. doi: 10.1007/s00415-020-10250-6 (PMC7914192; doi:10.1007/s00415-020-10250-6)
Supplement: Supplementary file 1 — Supplementary file1 (PDF 760 kb) [file 415_2020_10250_MOESM1_ESM.pdf]

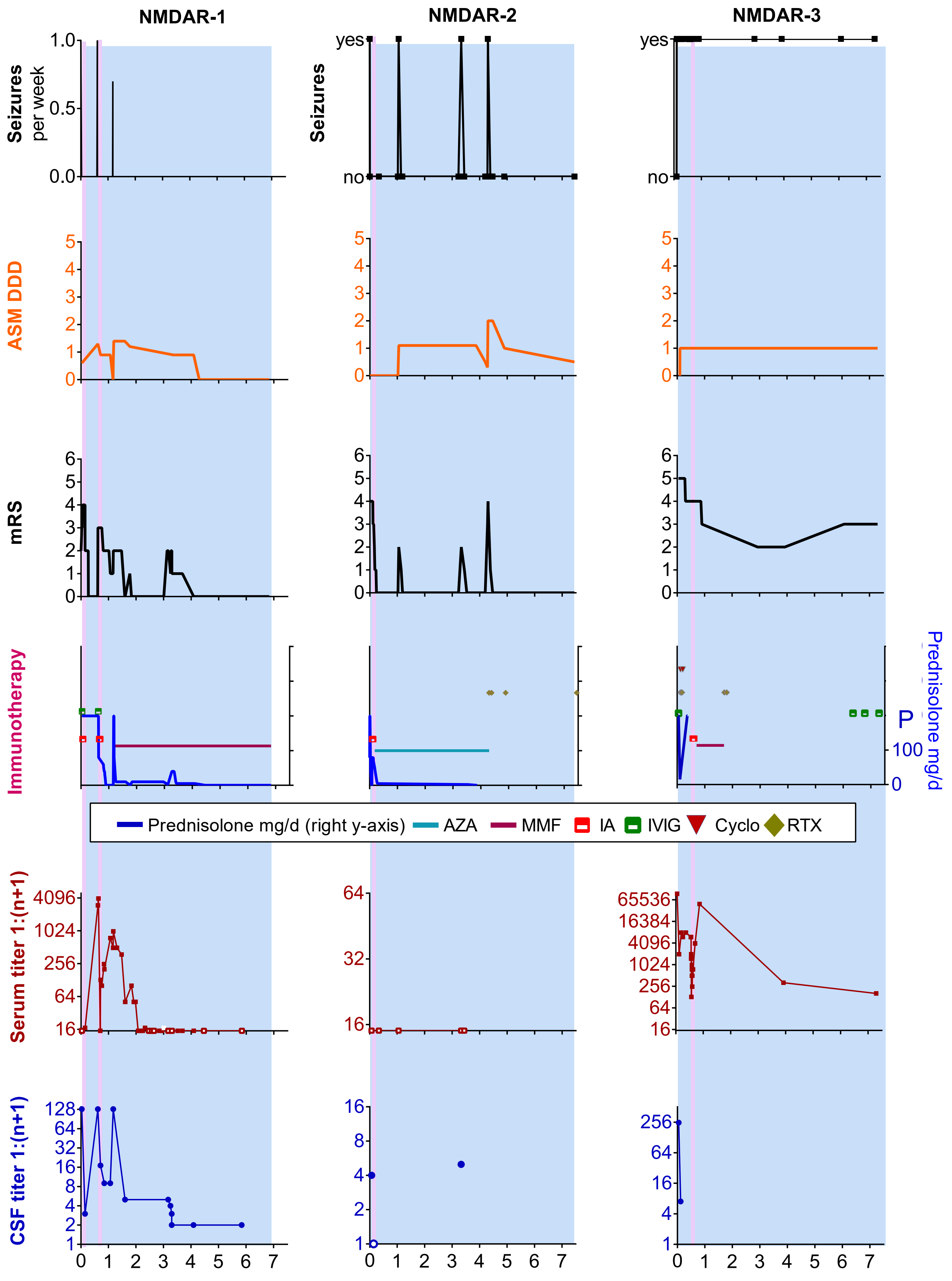

NMDAR-1
NMDAR-2
NMDAR-3
Seizures per week
Seizures
yes
no
ASM DDD
mRS
Immunotherapy
Prednisolone mg/d
P
Prednisolone mg/d (right y-axis)
AZA
MMF
IA
IVIG
Cyclo
RTX
Serum titer 1:(n+1)
CSF titer 1:(n+1)

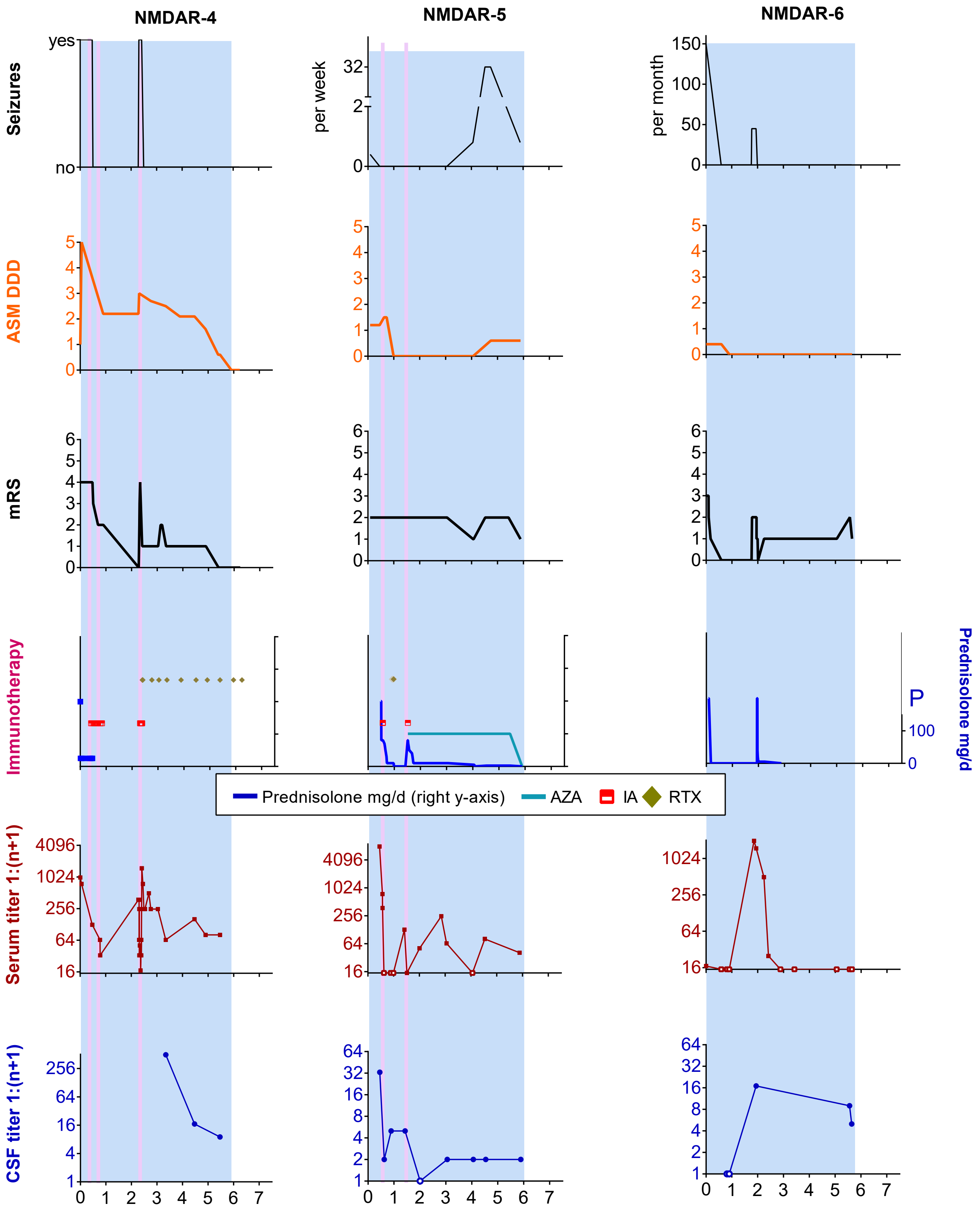

NMDAR-4
NMDAR-5
NMDAR-6
Seizures
yes
no
per week
per month
ASM DDD
mRS
Immunotherapy
Prednisolone mg/d
P
Prednisolone mg/d (right y-axis)
AZA
IA
RTX
Serum titer 1:(n+1)
CSF titer 1:(n+1)

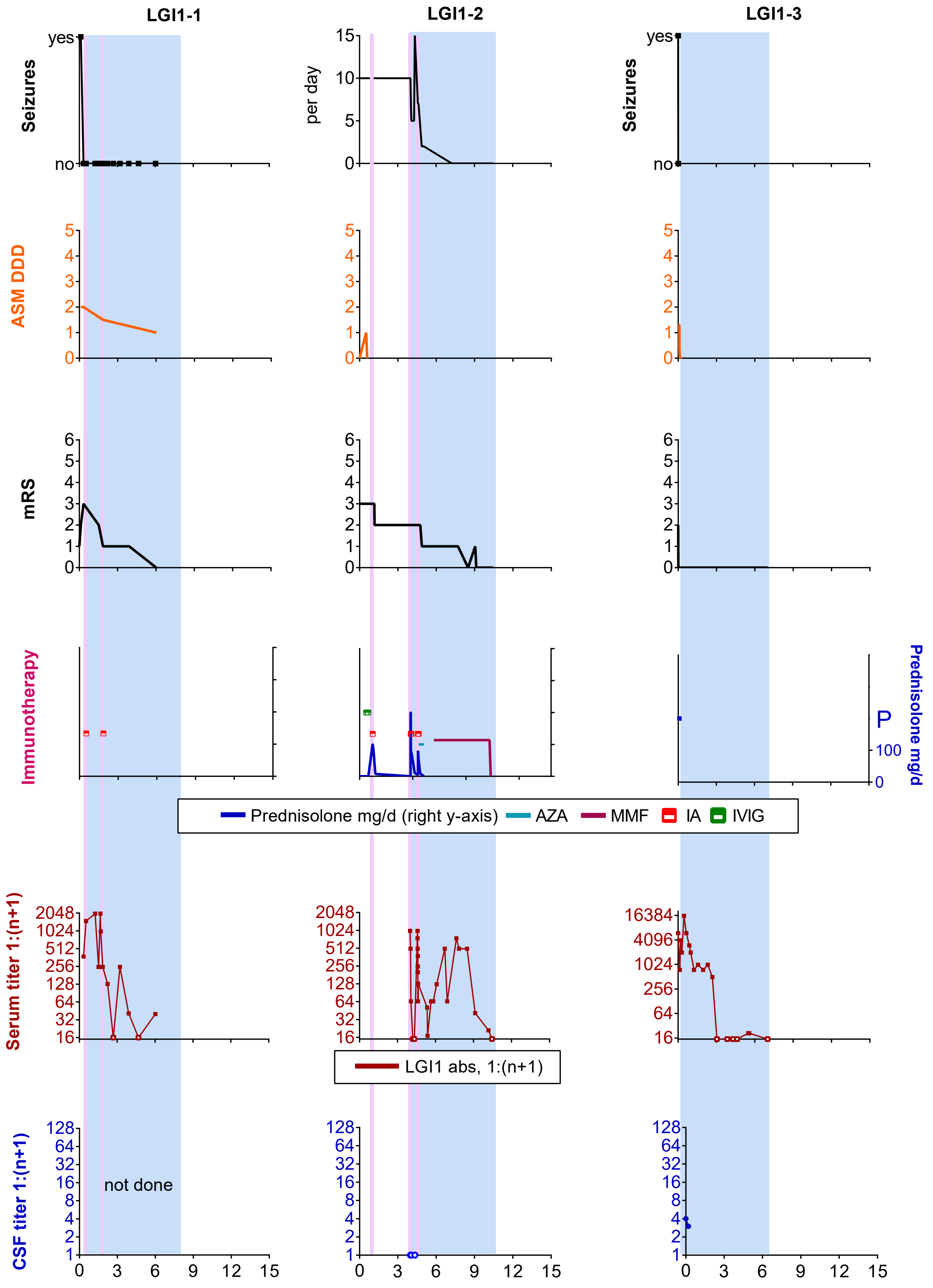
LGI1-1
LGI1-2
LGI1-3
Seizures
yes
no
per day
ASM DDD
mRS
Immunotherapy
Prednisolone mg/d
P
Prednisolone mg/d (right y-axis)
AZA
MMF
IA
IVIG
Serum titer 1:(n+1)
LGI1 abs, 1:(n+1)
CSF titer 1:(n+1)
not done

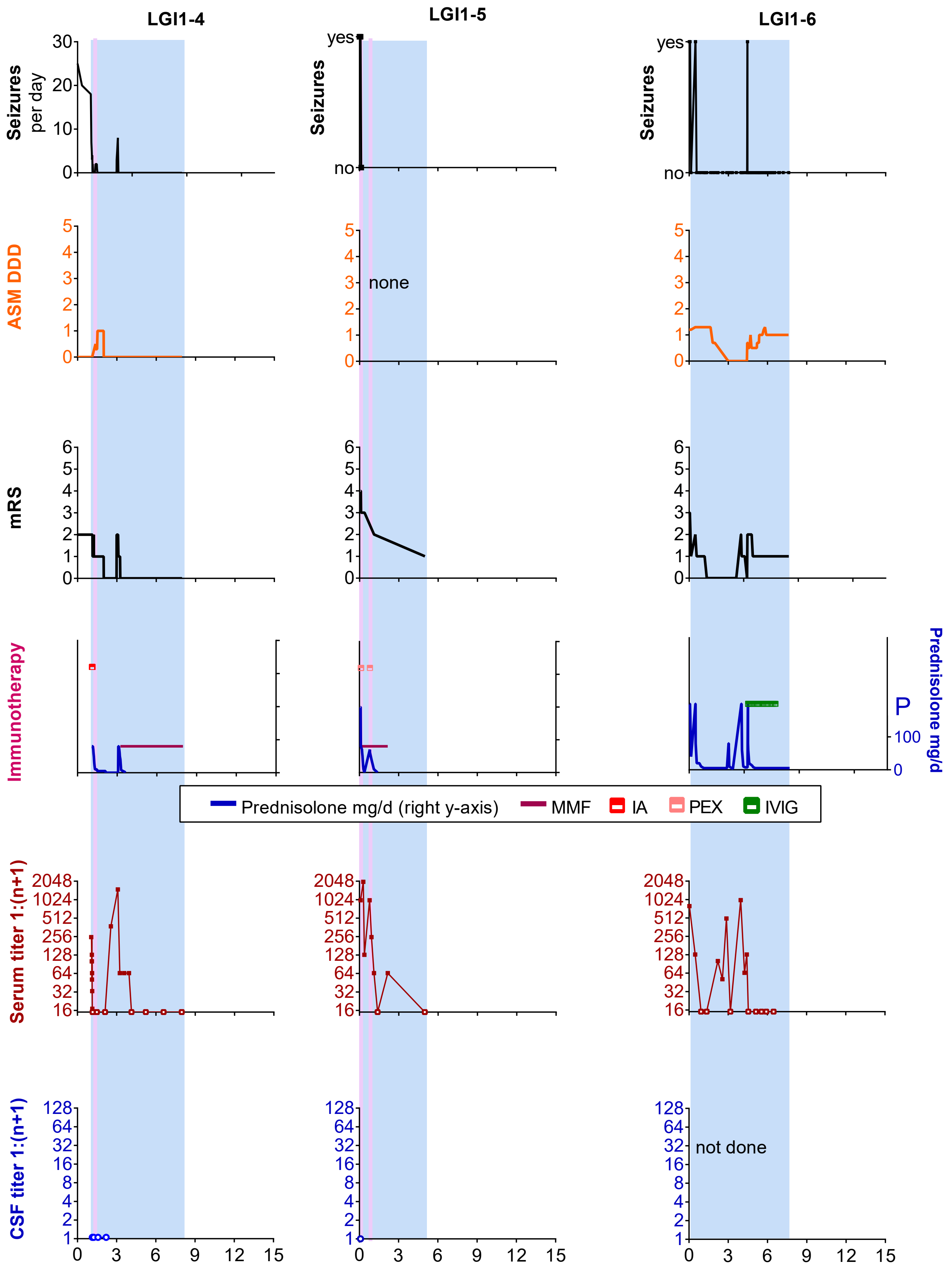

LGI1-4
LGI1-5
LGI1-6
Seizures per day
Seizures
yes
no
ASM DDD
none
mRS
Immunotherapy
P
Prednisolone mg/d
Prednisolone mg/d (right y-axis)
MMF
IA
PEX
IVIG
Serum titer 1:(n+1)
CSF titer 1:(n+1)
not done

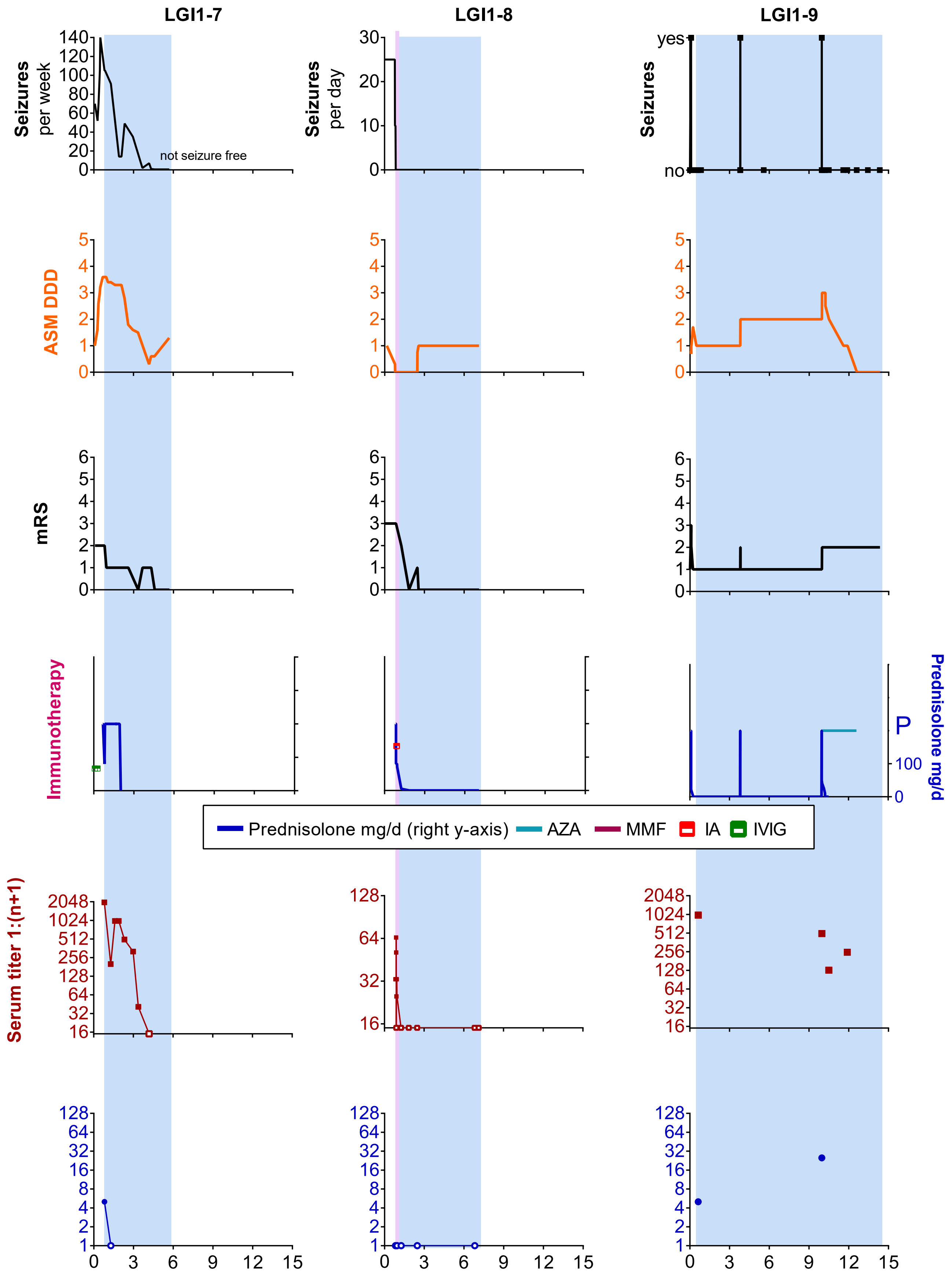

LGI1-7
LGI1-8
LGI1-9
Seizures per week
Seizures per day
Seizures
not seizure free
yes
no
ASM DDD
mRS
Immunotherapy
Prednisolone mg/d
P
100
Prednisolone mg/d (right y-axis)
AZA
MMF
IA
IVIG
Serum titer 1:(n+1)
0
3
6
9
12
15

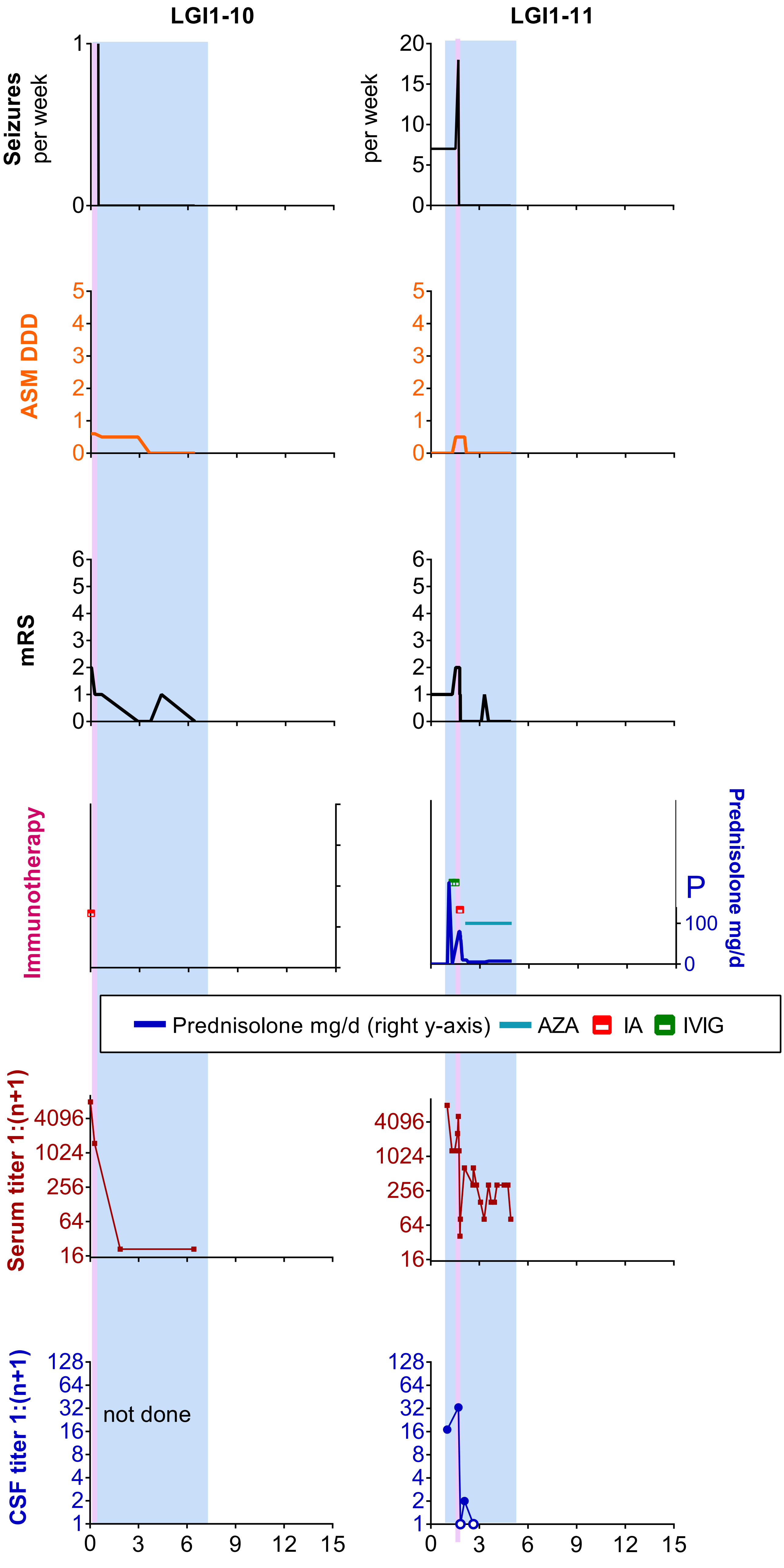

LGI1-10
LGI1-11
Seizures per week
per week
ASM DDD
mRS
Immunotherapy
P
Prednisolone mg/d
Prednisolone mg/d (right y-axis)
AZA
IA
IVIG
Serum titer 1:(n+1)
CSF titer 1:(n+1)
not done

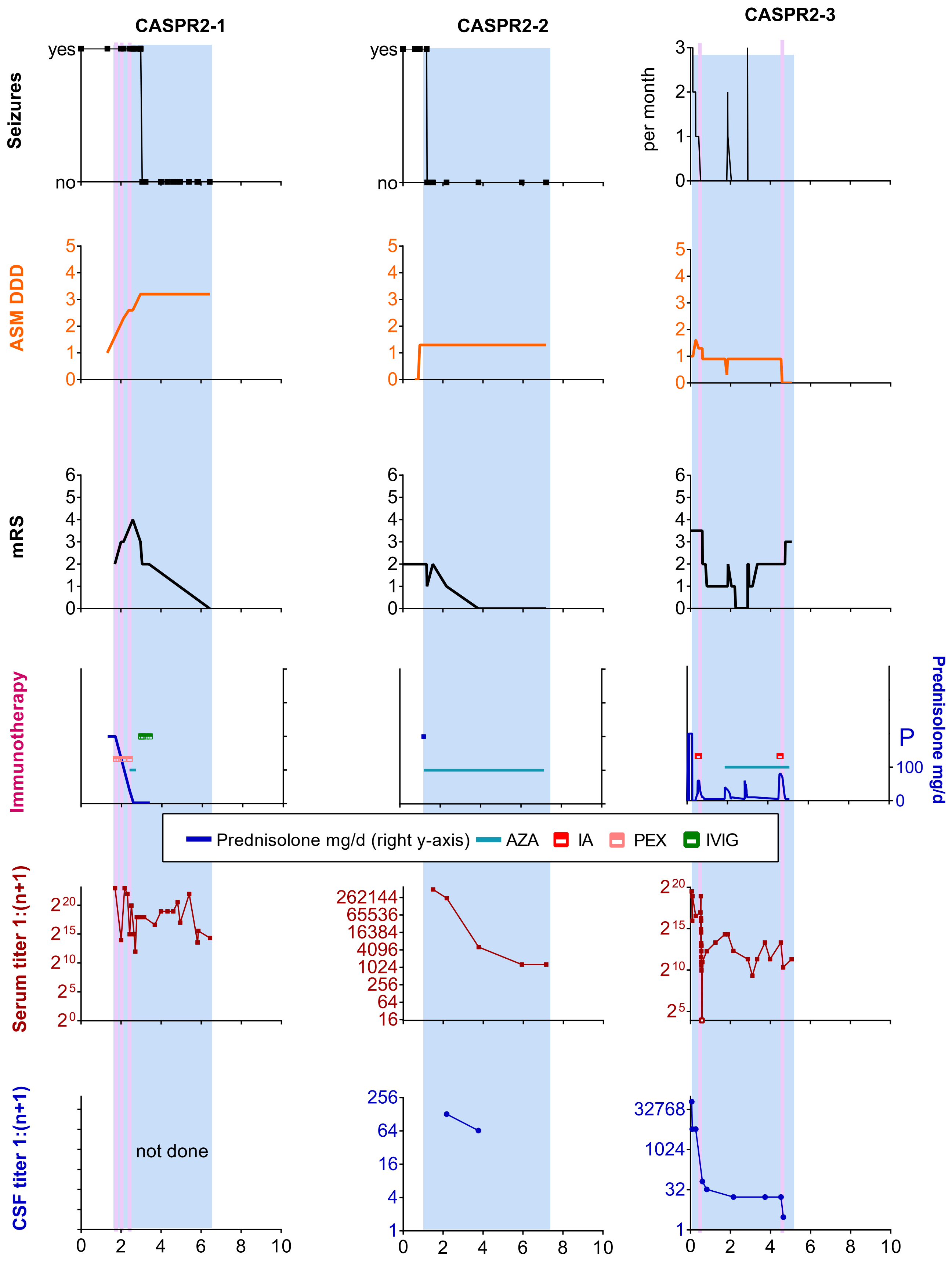

CASPR2-1
CASPR2-2
CASPR2-3
Seizures
yes
no
per month
ASM DDD
mRS
Immunotherapy
P
Prednisolone mg/d
Prednisolone mg/d (right y-axis)
AZA
IA
PEX
IVIG
Serum titer 1:(n+1)
262144
65536
16384
4096
1024
256
64
16
CSF titer 1:(n+1)
not done
256
64
16
4
1
32768
1024
32

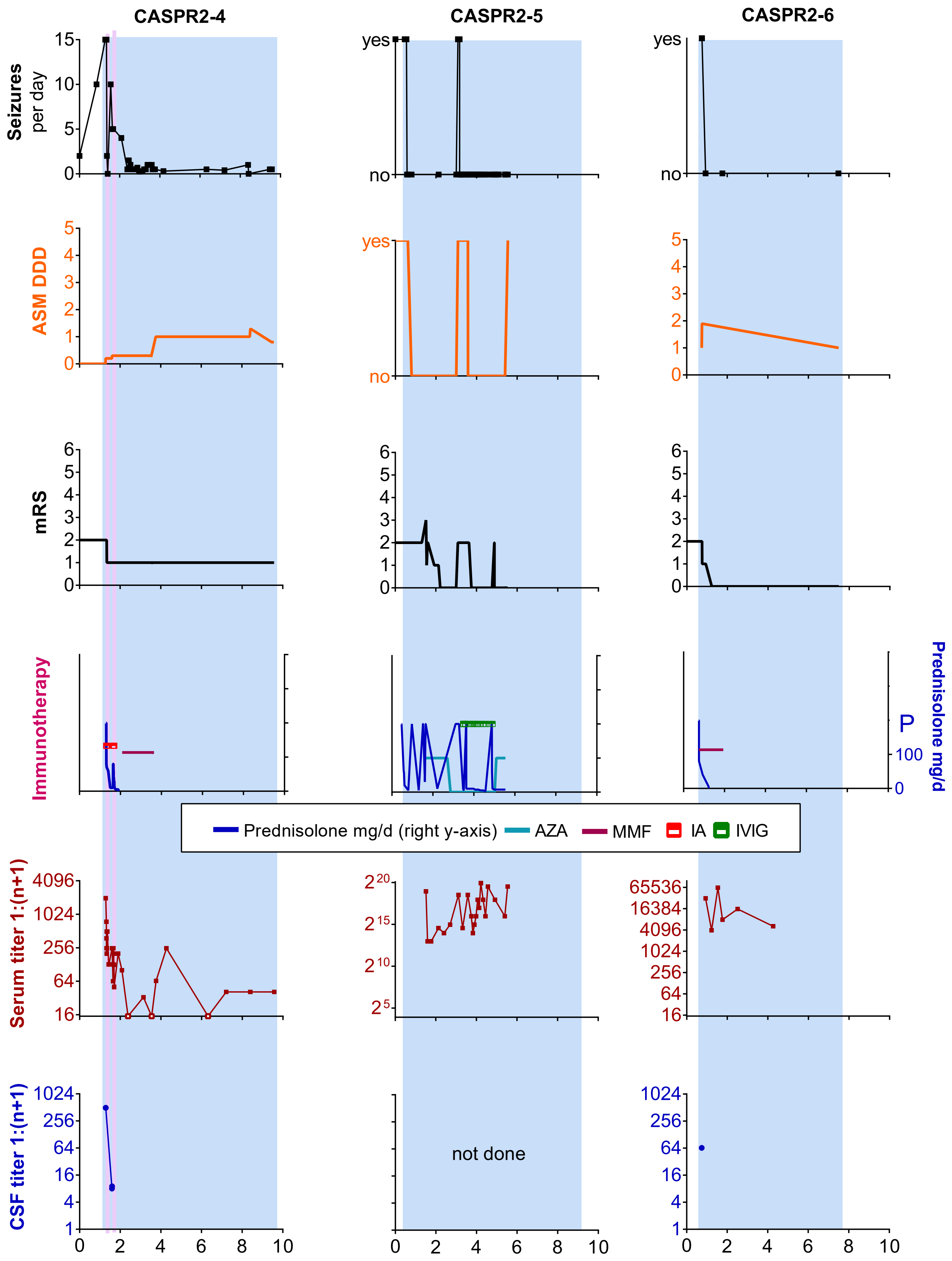

CASPR2-4
CASPR2-5
CASPR2-6
Seizures per day
yes
no
ASM DDD
mRS
Immunotherapy
Prednisolone mg/d
P
Prednisolone mg/d (right y-axis)
AZA
MMF
IA
IVIG
Serum titer 1:(n+1)
CSF titer 1:(n+1)
not done

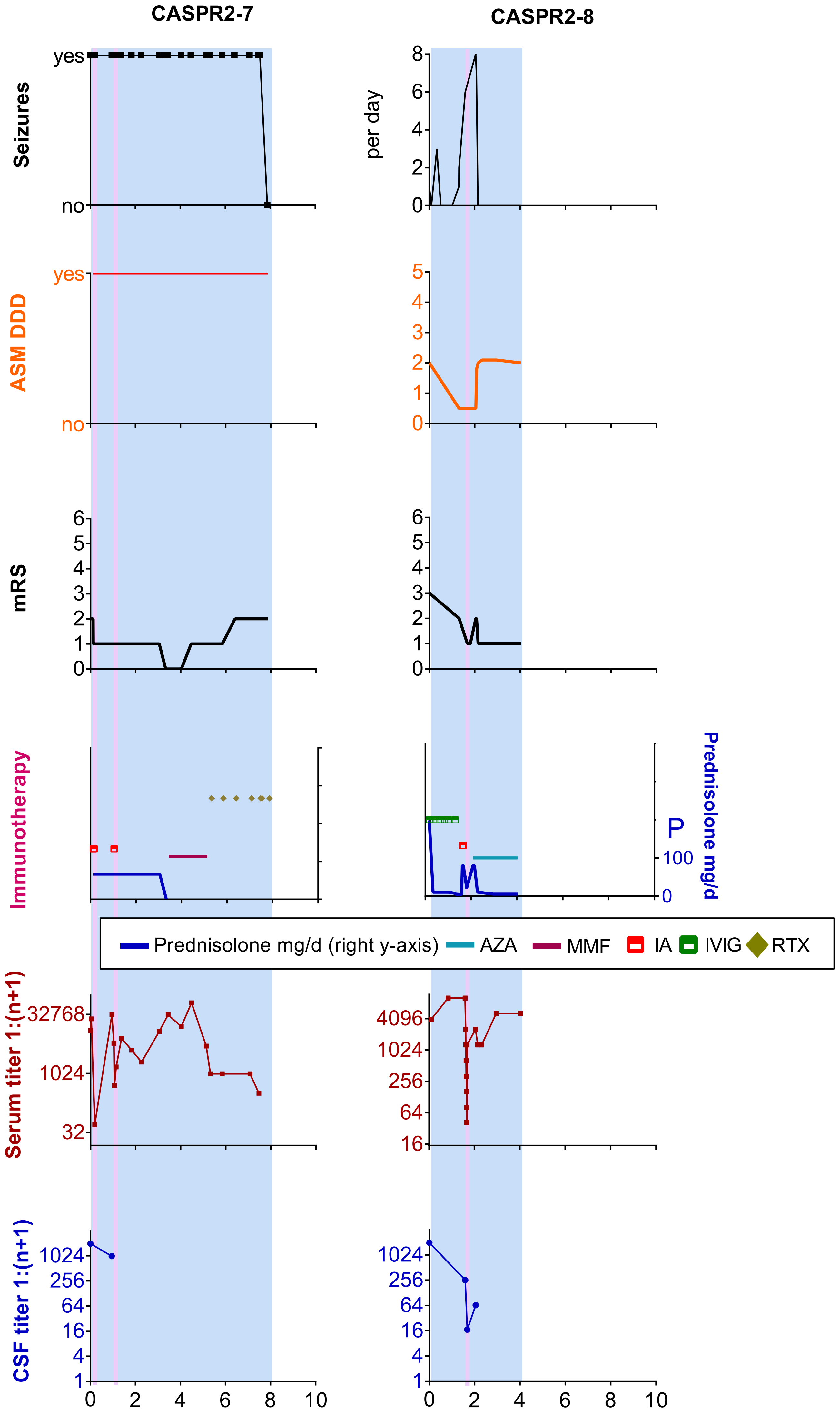
CASPR2-7
CASPR2-8
Seizures
yes
no
per day
ASM DDD
mRS
Immunotherapy
P
Prednisolone mg/d
Prednisolone mg/d (right y-axis)
AZA
MMF
IA
IVIG
RTX
Serum titer 1:(n+1)
CSF titer 1:(n+1)

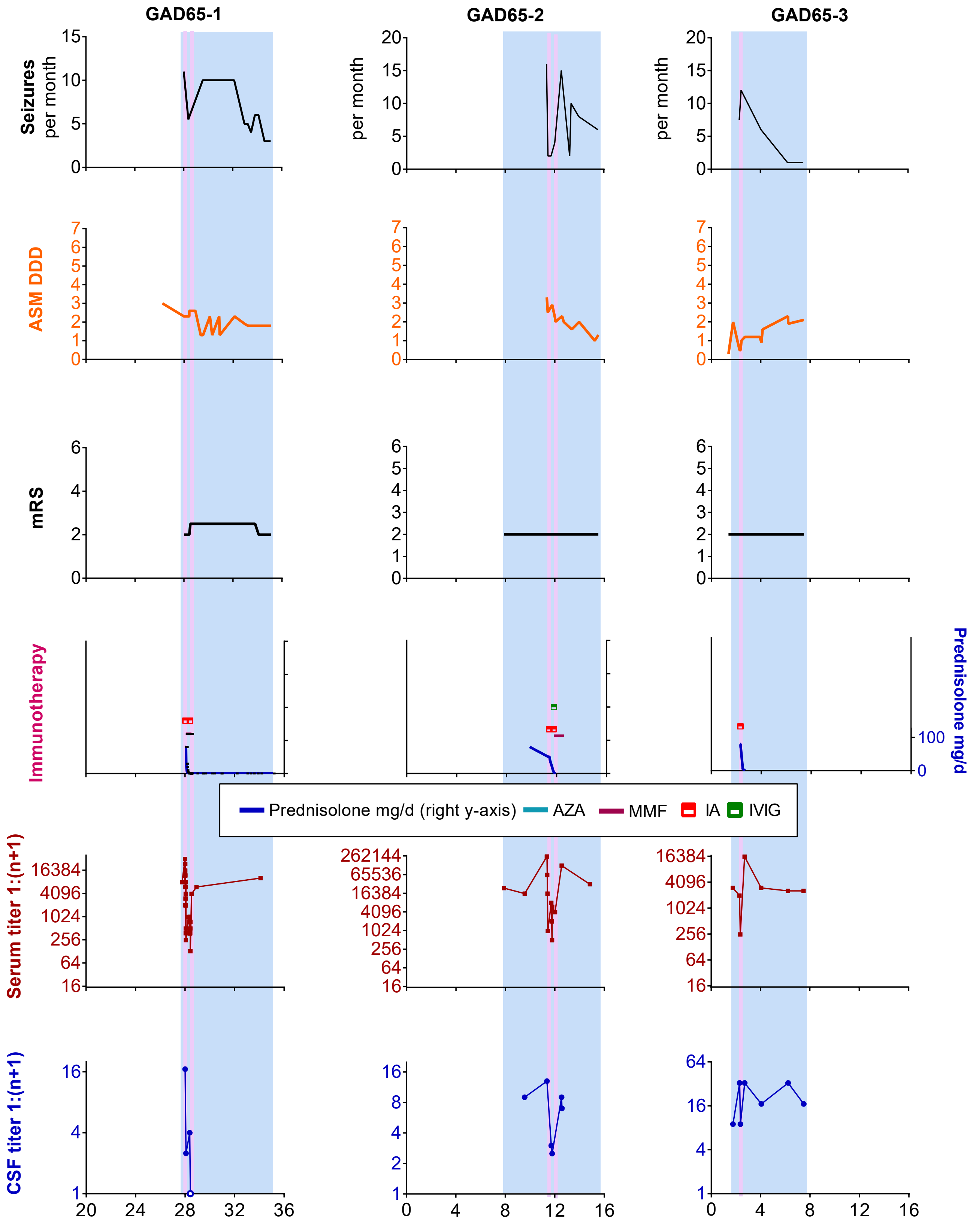

GAD65-1
GAD65-2
GAD65-3
Seizures per month
per month
per month
ASM DDD
mRS
Immunotherapy
Prednisolone mg/d
Prednisolone mg/d (right y-axis)
AZA
MMF
IA
IVIG
Serum titer 1:(n+1)
CSF titer 1:(n+1)

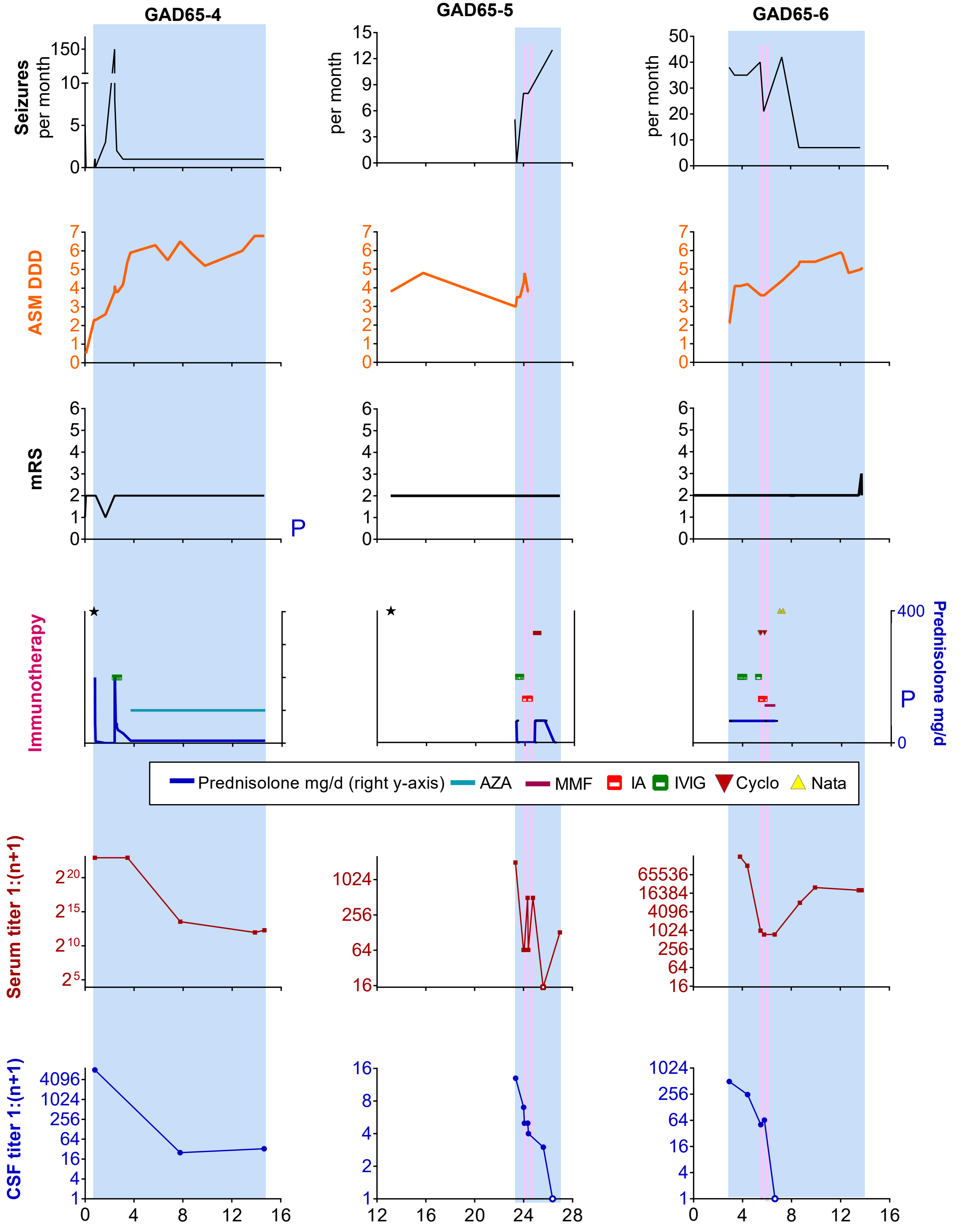

GAD65-4
GAD65-5
GAD65-6
Seizures per month
per month
ASM DDD
mRS
P
Immunotherapy
Prednisolone mg/d
Prednisolone mg/d (right y-axis)
AZA
MMF
IA
IVIG
Cyclo
Nata
Serum titer 1:(n+1)
CSF titer 1:(n+1)

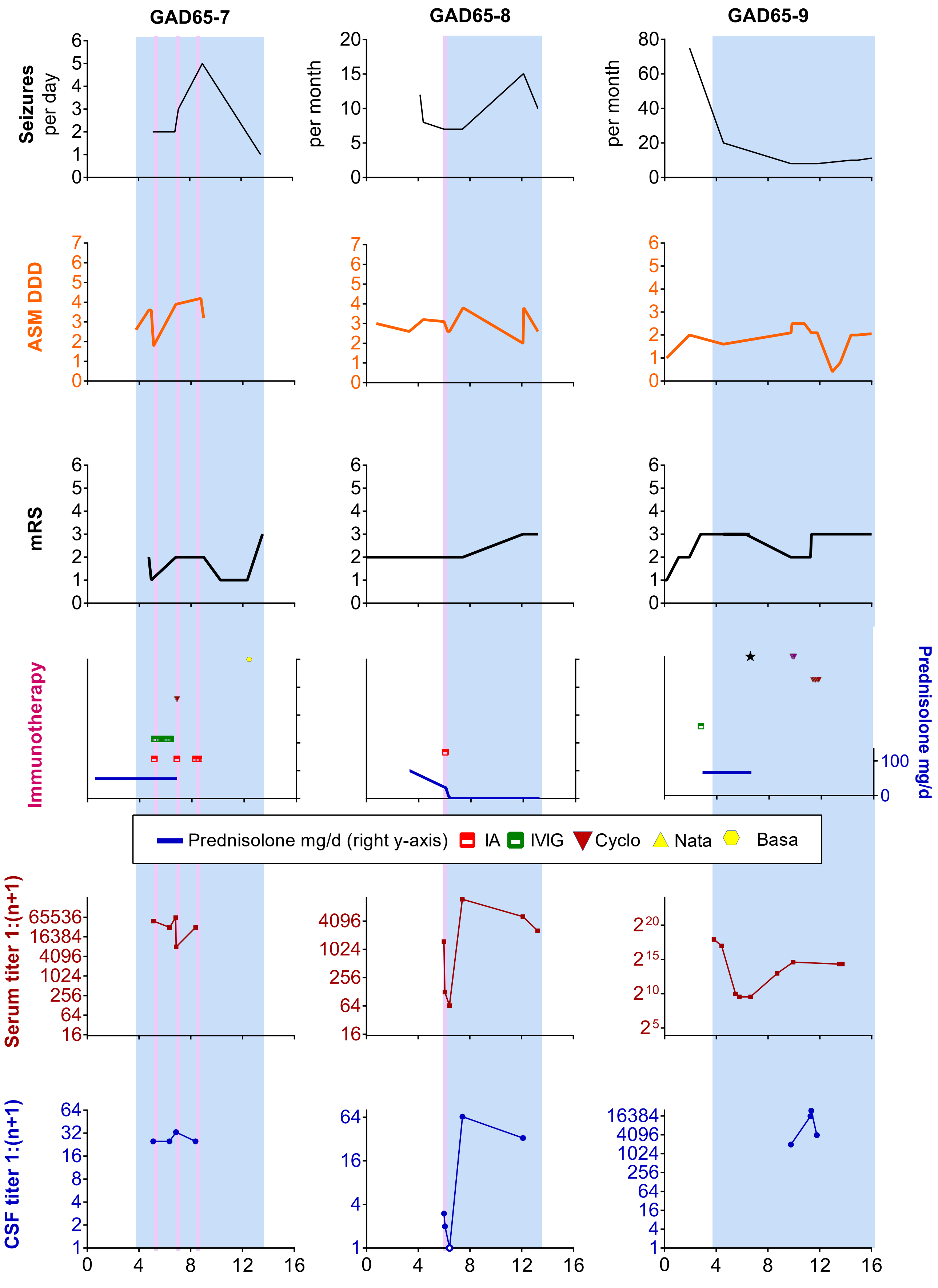
GAD65-7
GAD65-8
GAD65-9
Seizures per day
per month
per month
ASM DDD
mRS
Immunotherapy
Prednisolone mg/d
Prednisolone mg/d (right y-axis)
IA
IVIG
Cyclo
Nata
Basa
Serum titer 1:(n+1)
CSF titer 1:(n+1)

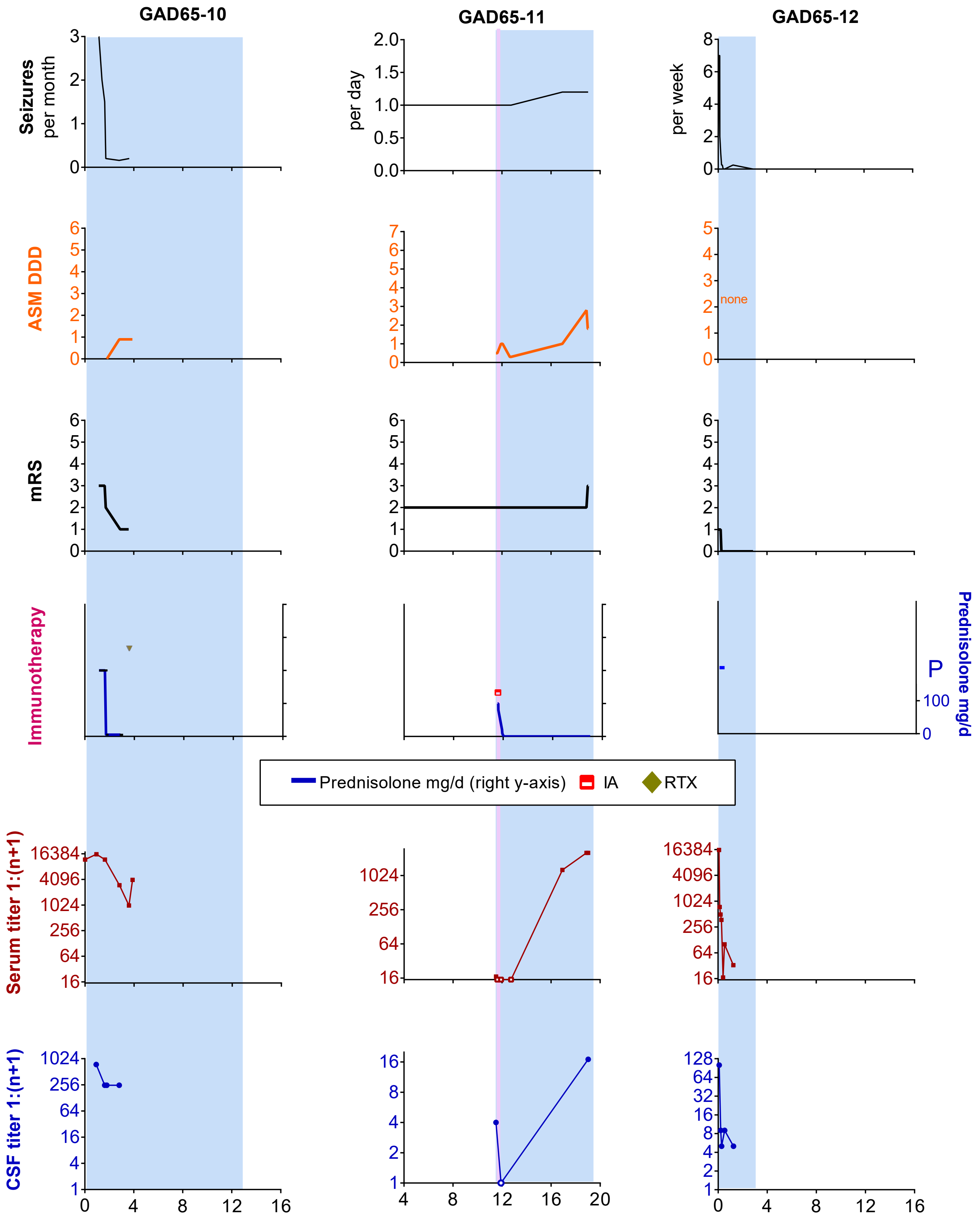
GAD65-10
GAD65-11
GAD65-12
Seizures per month
per day
per week
ASM DDD
none
mRS
Immunotherapy
P
Prednisolone mg/d
Prednisolone mg/d (right y-axis)
IA
RTX
Serum titer 1:(n+1)
CSF titer 1:(n+1)

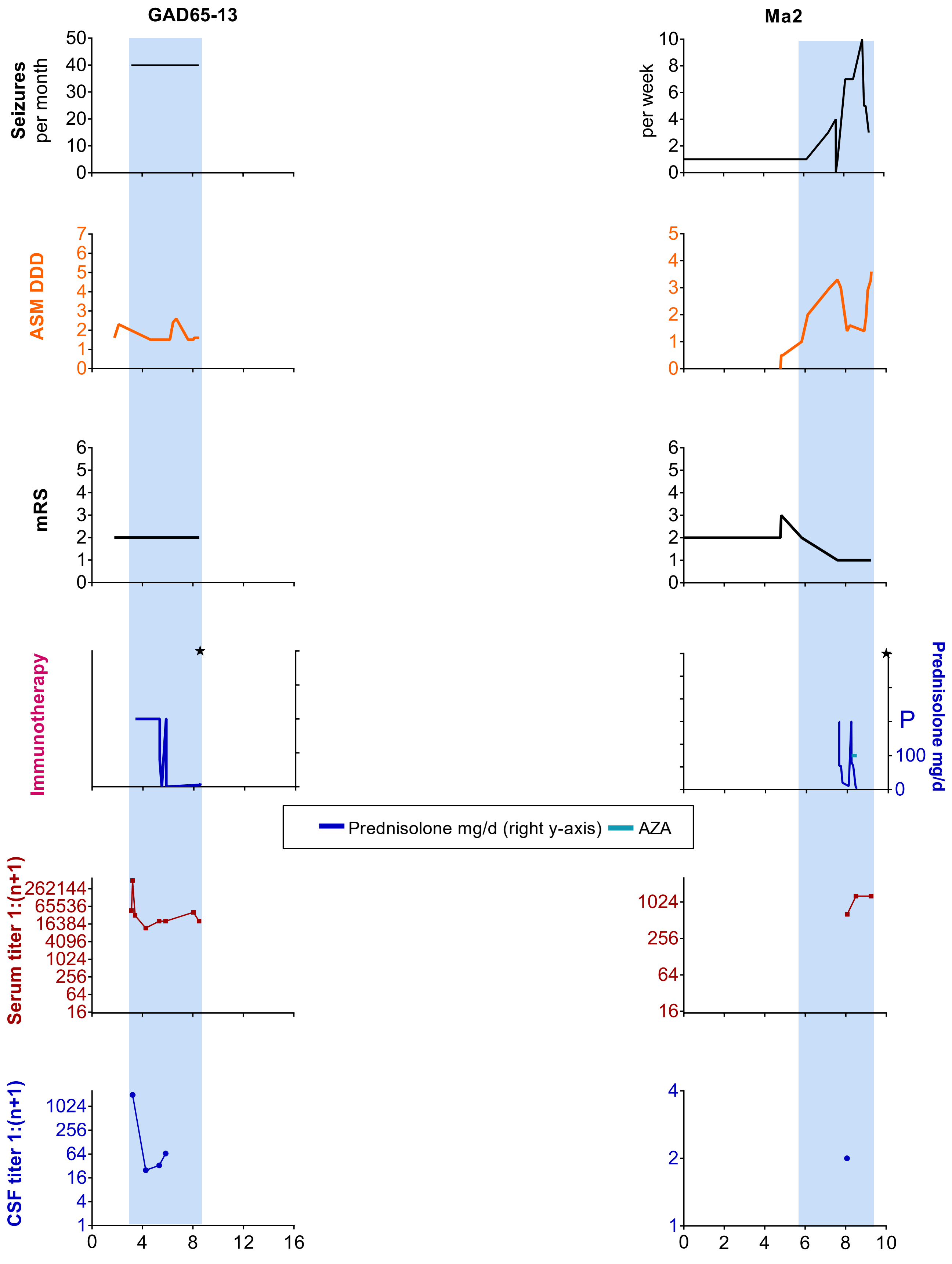

GAD65-13
Ma2
Seizures per month
per week
ASM DDD
mRS
Immunotherapy
Prednisolone mg/d
P
Prednisolone mg/d (right y-axis)
AZA
Serum titer 1:(n+1)
CSF titer 1:(n+1)

Supplementary Tables

Supplementary table 1: Individual patients' data

Refs, de-scrip-tive stati-stics	**Patient no., sex**	**Age at disease onset [y]**	**Time to antibody diagnosis [mo after onset]**	**ASM, maximum DDD**	**Time to start of first immuno-therapy [mo after onset]**	**No. of immuno-txs**	**Immunotherapies**	**Duration of immuno-therapy [mo][1]**	**No. of relapses**	**Time to last follow-up [mo after onset]**	**From ab detection to last follow-up [mo]**	**Time to first seizure freedom [mo after onset]**	**Time to terminal seizure freedom [mo after onset]**	**Duration of seizure freedom at most recent follow-up [mo]**	**Duration to discon-tinuation of ASM [mo]**
[4, 5]	NMDAR-1, m	14	0.4	1.4	0.4	4	Pred, IA, IVIG, MMF	53.6	5	82.0	81.6	14.1	14.1	67.9	51.5
[4, 5]	NMDAR-2, f	19	0	1.1	0	4	Pred, IA, Aza, Rtx	89.4[2]	3	89.4	89.4	0.1	41.0	36.7	Ongoing
[4]	NMDAR-3, f	2	0.6	1.5	0.6	6	Pred, IA, IVIG, Rtx, Cyc, MMF	87.0[2]	Never remitted	87.6	87.0	Ongoing	Ongoing	Ongoing	Ongoing
[4]	NMDAR-4, f	17	0.1	5	0	4	Pred, IA, Cyc, Rtx	74.9[2]	1	74.9	74.8	6.0	33.2	41.7	Ongoing
[4, 5]	NMDAR-5, f	18	5.4	1.5	5.9	4	Pred, IA, Rtx, Aza	64.8	Never remitted	70.7	65.3	Ongoing	Ongoing	Ongoing	Ongoing
[4]	NMDAR-6, f	6	0	0.4	1	2	Pred	33.5	1	67.7	67.7	7.1	23.9	43.8	10.8
**Med**	m/f: 1/5	**16**	**0.3**	**1.5**	**0.5**	**4**		**69.9**	**2**	**78.5**	**78.2**	**6.6**	**28.5**	**42.8**	**31.2**
**Min**		**2**	**0.0**	**0.4**	**0.0**	**2**		**33.5**	**1**	**67.7**	**65.3**	**0.1**	**14.1**	**36.7**	**10.8**
**Max**		**19**	**5.4**	**5.0**	**5.9**	**6**		**89.4**	**5**	**89.4**	**89.4**	**14.1**	**41.0**	**67.9**	**51.5**
[4]	LGI1-1, m	73	4	2	6.3	1	IA	15.9	0	72.3	68.3	4.0	4.0	70.8	Ongoing
[4, 5]	LGI1-2, m	59	47.9	1	6.1	5	Pred, Aza, MMF, IA, IVIG	117.2	0	125.5	77.6	86.7	86.7	38.8	7.2
[4]	LGI1-3, f	51	0.1	0	1.1	1	Pred	0.2	0	84.1	84.0	0.2	0.2	84	1.7
[4, 7]	LGI1-4, f	65	12.7	1.3	13	4	Pred, Aza, MMF, IA	82.6[2]	1	95.6	82.9	18.3	37.4	58.2	24
[4, 8]	LGI1-5, m	14	1	0	1.1	3	Pred, PEX, MMF	15.6	0	59.8	58.8	1.6	1.6	58.2	Never had ASM
[4]	LGI1-6, m	76	0.4	0	0.6	3	Pred, Aza, IVIG	90.7[2]	1	91.3	90.9	6.9	53.5	53.5	Ongoing
[4, 5]	LGI1-7, m	73	9.7	1.3	8.2	3	Pred, IVIG, Aza	16	Never remitted	68.2	58.5	Ongoing	Ongoing	Ongoing	Ongoing
[4, 5]	LGI1-8, m	50	9.9	3.6	10.1	2	Pred, Aza	12	0	85.5	75.6	10.2	10.2	75.3[3]	Ongoing

Refs, descriptive statistics	Patient no., sex	Age at disease onset [y]	Time to antibody diagnosis [mo after onset]	ASM, maximum DDD	Time to start of first immunotherapy [mo after onset]	No. of immunotxs	Immunotherapies	Duration of immunotherapy [mo][1]	No. of relapses	Time to last follow-up [mo after onset]	From ab detection to last follow-up [mo]	Time to first seizure freedom [mo after onset]	Time to terminal seizure freedom [mo after onset]	Duration of seizure freedom at most recent follow-up [mo]	Duration to discontinuation of ASM [mo]
[4]	LGI1-9, m	67	7.6[4]	1	1	2	Pred, Aza	150.2	2	172.0	164.4	1.2	119.7	52.5	151.2
[4]	LGI1-10, m	49	0.1	2.5	0.4	1	IA	0.2	0	77.0	76.9	8.0	8.0	71.3	44
[4]	LGI1-11, f	65	12.2	0.6	13.2	4	Pred, IA, IVIG, Aza	46.1[2]	0	59.3	47.1	21.0	21.0	38.3	26.2
**Med**	m/f: 8/3	**65**	**7.6**	**1.0**	**6.1**	**3**		**16.0**	**0**	**84.1**	**76.9**	**7.4**	**15.6**	**58.2**	**25.1**
**Min**		**14**	**0.1**	**0.0**	**0.4**	**1**		**0.2**	**0**	**59.3**	**47.1**	**0.2**	**0.2**	**38.3**	**1.7**
**Max**		**76**	**47.9**	**3.6**	**13.2**	**5**		**150.2**	**2**	**172.0**	**164.4**	**86.7**	**119.7**	**84.0**	**151.2**
[3, 4]	CASPR2-1, m	75	20.3[4]	**3.6**	15.8	4	Pred, Aza, IA, IVIG	28.4	0	77.3	57.0	37.0	37.0	40.5	Ongoing
[3, 4]	CASPR2-2, m	56	17.6[4]	3.2	14.1	2	Pred, Aza	71.6[2]	0	85.7	68.1	14.5	14.5	71.3	Ongoing
[4]	CASPR2-3, m	75	0.8[4]	1.3	0.6	3	Pred, IA, Aza	55.1[2]	2	55.7	54.9	6.0	34.4	21.2	54.9
[3-5]	CASPR2-4, m	69	15.4	1.6	15.5	3	Pred, IA, MMF	28.1	0	114.9	99.5	Ongoing	Ongoing	Ongoing	Ongoing
[3, 4]	CASPR2-5, m	68	18.2[4]	1.3	5.6	3	Pred, Aza, IVIG	60.9[2]	2	66.5[5]	48.3	6.9	38.0	28.5	Ongoing
[3, 4]	CASPR2-6, m	69	8.9	Unknown	8.9	2	Pred, MMF	14.4	0	89.8	80.9	11.2	11.2	78.7	Ongoing
[3, 4]	CASPR2-7, m	70	0	1.9	1.4	4	Pred, IA, MMF, Rtx	38.5	Never remitted	94.4	94.4	Ongoing	Ongoing	Ongoing	Ongoing
[4]	CASPR2-8, m	49	1.2	Unknown	2	4	Pred, Aza, IA, IVIG	46.3[2]	1	48.3	47.1	25.8	25.8	22.7	Ongoing
**Med**	m/f: 8/0	**69**	**12.2**	1.75	**7.3**	**3**		**42.4**	**0**	**81.5**	**62.5**	**12.8**	**30.1**	**34.5**	**54.9**
**Min**		**49**	**0.0**	**1.3**	**0.6**	**2**		**14.4**	**0**	**48.3**	**47.1**	**6.0**	**11.2**	**21.2**	**54.9**
**Max**		**75**	**20.3**	**3.6**	**15.8**	**4**		**71.6**	**2**	**114.9**	**99.5**	**37.0**	**38.0**	**78.7**	**54.9**
[4, 5]	GAD65-1, f	31	333.1		336	3	Pred, IA, MMF	6.2	Never remitted	421.2	88.1	421.2	Ongoing	Ongoing	Ongoing
[4-6]	GAD65-2, f	24	94.6	3	118.1	4	Pred, MMF, IA, IVIG	32.8	Never remitted	186.3	91.7	186.3	Ongoing	Ongoing	Ongoing
[4]	GAD65-3, m	22	21.1	3.3	27.7	2	Pred, IA	4.8	Never remitted	89.8	68.7	89.8	Ongoing	Ongoing	Ongoing
[1, 4, 6]	GAD65-4, f	23	9.5	2.3	10	3	Pred, Aza, IVIG	165.7[2]	Never remitted	175.7	166.2	175.0	Ongoing	Ongoing	Ongoing

Refs, de-scrip-tive stati-stics	Patient no., sex	Age at disease onset [y]	Time to antibody diagnosis [mo after onset]	ASM, maximum DDD	Time to start of first immuno-therapy [mo after onset]	No. of immuno-txs	Immunotherapies	Duration of immuno-therapy [mo][1]	No. of relapses	Time to last follow-up [mo after onset]	From ab detection to last follow-up [mo]	Time to first seizure freedom [mo after onset]	Time to terminal seizure freedom [mo after onset]	Duration of seizure freedom at most recent follow-up [mo]	Duration to discon-tinuation of ASM [mo]
2, 4, 6	GAD65-5, f	8	280.1[4]	6.8	279.5	4	Pred, IA, IVIG, Cyc	36.9	Never remitted	323.8	43.7	323.8	Ongoing	Ongoing	Ongoing
4, 6	GAD65-6, f	15	35	4.8	35	6	Pred, MMF, IA, IVIG, Cyc, Natalizumab	52.3	Never remitted	165.3	130.3	165.3	Ongoing	Ongoing	Ongoing
4, 6	GAD65-7, f	21	61.2[4]	5.8	7.1	7	Pred, Aza, IA, IVIG, Rtx, Cyc, Basiliximab	141.5	Never remitted	162.3	101.1	162.3	Ongoing	Ongoing	Ongoing
4, 5	GAD65-8, f	31	72	4.2	39.6	2	Pred, IA	37.4	Never remitted	158.9	86.9	158.9	Ongoing	Ongoing	Ongoing
4	GAD65-9, f	15	117.3[4]	3.8	13.2	4	Pred, IVIG, Cyc, Natalizumab	128.5	Never remitted	200.1	82.8	200.1	Ongoing	Ongoing	Ongoing
4	GAD65-10, f	7	0	2.5	13.8	2	Pred, Rtx,	29.7	Never remitted	46.5	46.5	46.5	Ongoing	Ongoing	Ongoing
4, 5	GAD65-11, f	23	138.1	3	138.6	2	Pred, IA	6	Never remitted	228.3	90.2	228.3	Ongoing	Ongoing	Ongoing
4	GAD65-12, f	15	1	2.8	1.5	1	Pred	5	0	36.4	36.4	4.0	4.0	32.4	Never had ASM
4	GAD65-13, f	16	37.6	0	40.9	1	Pred	61.4[2]	Never remitted	102.3	Ongoing	101.9	Ongoing	Ongoing	Ongoing
**Med**	m/f: 1/12	**21**	**61.2**	3.15	**35.0**	**3**		**36.9**	**0**	**165.3**	**87.5**	**165.3**	**5.0**	**31.4**	-
**Min**		**7**	**0.0**	**0.0**	**1.5**	**1**		**4.8**	**0**	**36.4**	**36.4**	**5.0**	**5.0**	**31.4**	-
**Max**		**31**	**333.1**	**6.8**	**336.0**	**7**		**165.7**	**0**	**421.2**	**166.2**	**421.2**	**5.0**	**31.4**	-
	**Ma2, m**	25	91.1	2.9	91.2	2	Pred, Aza	20		111.2	41.2	Ongoing	Ongoing	Ongoing	Ongoing

[1] Duration from first to final dose (intervals without immunotherapy included). [2]Duration is identical with difference between "last follow-up" and "start of immunotherapy", i.e., immunotherapy was ongoing at most recent follow-up. [3]One subclinical seizure pattern recorded on long-term EEG 82.8 months after disease onset. Not counted here. [4]First titer in this laboratory; probably, the antibody diagnosis was made in an external lab earlier on. [5]Died from liver failure.

Abbreviations: ASM, anti-seizure medication; Aza, azathioprine; Cyc, cyclophosphamide; DDD, defined daily doses; f, female; IA, immunoadsorption; IVIG, intravenous immunoglobulins; m, male; Max, maximum; Med, median; Min, minimum; MMF, mycophenolate-mofetil; mo, months; No., number; PEX, plasma exchange; Pred, oral prednislolone or intravenous methylprednisolone; Refs, references including the respective case (usually with shorter observational period); Rtx, rituximab; y, years

Supplementary table 2: Year-wise proportion of seizure free patients

Delay (years)	NMDAR antibodies		LGI1 antibodies*		CASPR2 antibodies		GAD/Ma2 antibodies**		Ma2 antibodies	
	First seizure freedom	Terminal seizure freedom	First seizure freedom	Terminal seizure freedom	First seizure freedom	Terminal seizure freedom	First seizure-freedom	Terminal seizure freedom	First seizure freedom	Terminal seizure freedom
1	67%	0%	64%	46%	38%	13%	8%	8%	8mo]0%	0%
2	83%	33%	82%	55%	50%	25%	8%	8%	0%	0%
3	83%	50%	82%	55%	63%	50%	8%	8%	0%	0%
4	83%	67%	82%	64%	75%	75%	8%	8%	0%	0%

*After 10 years: 91% terminal seizure freedom; **only one patient with GAD65 antibodies became seizure free (within 4 months).

Supplementary table 3: Patients achieving first seizure freedom after >1 year

Patient	First seizure stop			Terminal seizure stop				Terminal ASM freedom	
	Time to [mo]	Modification of immuno-tx	Modification of ASM	Time to [mo]	Modification of immuno-tx	Modification of ASM	Duration of terminal seizure freedom at most recent follow-up [mo]	Time to terminal ASM freedom	Duration of ASM freedom at most recent follow-up
Rapid relapses prior to first seizure freedom									
NMDAR-1	14	Steroids introduced again plus MMF	Increase from shortly discontinued ASM to 0.7 DDD	= first seizure stop			68	52	31
CASPR2-8	26	Steroids introduced again plus MMF	From 0.5 to 2.0 DDD	= first seizure stop			23	Ongoing ASM	-
Continuously recurrent seizures prior to first seizure freedom									
LGI1-2	87	MMF	None[1]	= first seizure stop			39	7	118
LGI1-4	18	Steroids introduced again	Introduction of ASM, 1.0 DDD	37	Steroids introduced again plus MMF	None[2]	58	24	72
LGI1-11	21	Steroids introduced again plus IA	None	= first seizure stop			38	26	33
CASPR2-1	37	IVIG	From 2.5 to 3.0 DDD	= first seizure stop			41	Ongoing ASM	-
CASPR2-2	15	Steroids	Introduction of ASM, 1.3 DDD	= first seizure stop			71	Ongoing ASM	-

[1]ASM stopped 80 mo before by patient's decision; [2]ASM stopped 13 mo before by patient's decision

Abbreviations: ASM, anti-seizure medication; CASPR2, contactin-associated protein-2; DDD, defined daily doses (according to World Health Organization); IA, immunoadsorption; immuno-tx, immunotherapy; IVIG, intravenous immunoglobulins; LGI1, leucine-rich glioma inactivated protein 1; MMF, mycophenolate-mofetil; mo, months; NMDAR, N-methyl-D-aspartate receptor

References

1. Bien CG, Urbach H, Schramm J, Soeder BM, Becker AJ, Voltz R, et al. Limbic encephalitis as a precipitating event in adult-onset temporal lobe epilepsy. Neurology. 2007; 69: 1236-44.

2. Bien CG, Vincent A, Barnett MH, Becker AJ, Blümcke I, Graus F, et al. Immunopathology of autoantibody-associated encephalitides: clues for pathogenesis. Brain. 2012; 135: 1622-38.

3. Bien CG, Mirzadjanova Z, Baumgartner C, Onugoren MD, Grunwald T, Holtkamp M, et al. Anti-contactin-associated protein-2 encephalitis: relevance of antibody titres, presentation and outcome. Eur J Neurol. 2017; 24: 175-86.

4. Bien CG, Bien CI, Dogan Onugoren M, De Simoni D, Eigler V, Haensch C-A, et al. Routine diagnostics for neural antibodies, clinical correlates, treatment and functional outcome. J Neurol. 2020; in press.

5. Dogan Onugoren M, Golombeck KS, Bien C, Abu-Tair M, Brand M, Bulla-Hellwig M, et al. Immunoadsorption therapy in autoimmune encephalitides. Neurol Neuroimmunol Neuroinflamm. 2016; 3: e207.

6. Malter MP, Frisch C, Zeitler H, Surges R, Urbach H, Helmstaedter C, et al. Treatment of immune-mediated temporal lobe epilepsy with GAD antibodies. Seizure. 2015; 30: 57-63.

7. Scherfeld D, Bien CG. Antikörper-vermittelte Epilepsien. Klin Neurophys. 2014; 45: 222-9.

8. Schimmel M, Frühwald MC, Bien CG. Limbic encephalitis with LGI1 antibodies in a 14-year-old boy. Eur J Paediatr Neurol. 2018; 22: 190-3.
